# Supplementary material for: MAPP unravels frequent co-regulation of splicing and polyadenylation by RNA-binding proteins and their dysregulation in cancer
Source: Nat Commun. 2024 May 15;15:4110. doi: 10.1038/s41467-024-48046-1 (PMC11096328; doi:10.1038/s41467-024-48046-1)
Supplement: Supplementary file 1 — Supplementary Information [file 41467_2024_48046_MOESM1_ESM.pdf]

Supplementary Information

Maciej Bak[1,2*], Erik van Nimwegen[1,2], Ian U. Kouzel[3], Tamer Gur[3], Ralf Schmidt[1,2], Mihaela Zavolan[1,2] and Andreas J. Gruber[3*§]

1 Swiss Institute of Bioinformatics, 1015 Lausanne, Switzerland
2 Biozentrum, University of Basel, 4056 Basel, Switzerland
3 Department of Biology, University of Konstanz, D-78464 Konstanz, Germany
∗ These authors made equal first author contributions
§ To whom correspondence should be addressed: gruber@uni-konstanz.de

1 Supplementary Figures

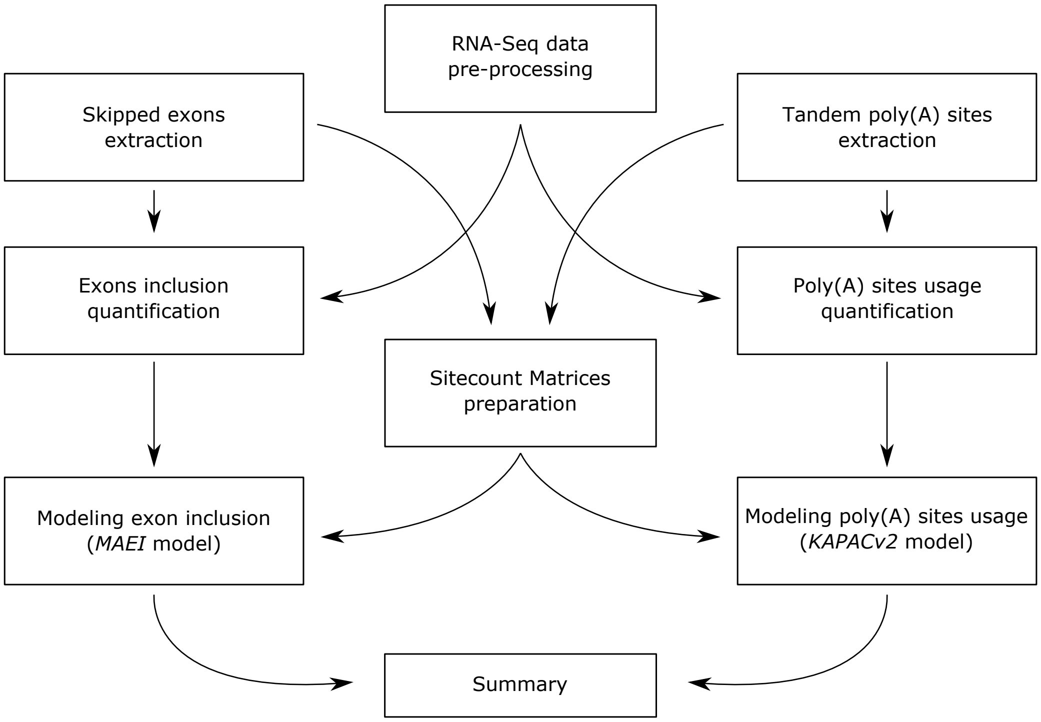

Figure S1. High-level overview of MAPP. The pipeline may be decomposed into nine separate functional sub-modules, each of which can be executed individually on its own. Modules on the left-hand side are related to the analysis of alternative splicing, whereas the right-hand side is dedicated to alternative polyadenylation. The modules in the middle preprocess and prepare the RNA-seq data for the splicing and the polyadenylation modules (top), create the sitecount matrixes (middle) and summarize the results in the form of a report (bottom). Accordingly, MAPP starts from three independent entry points.

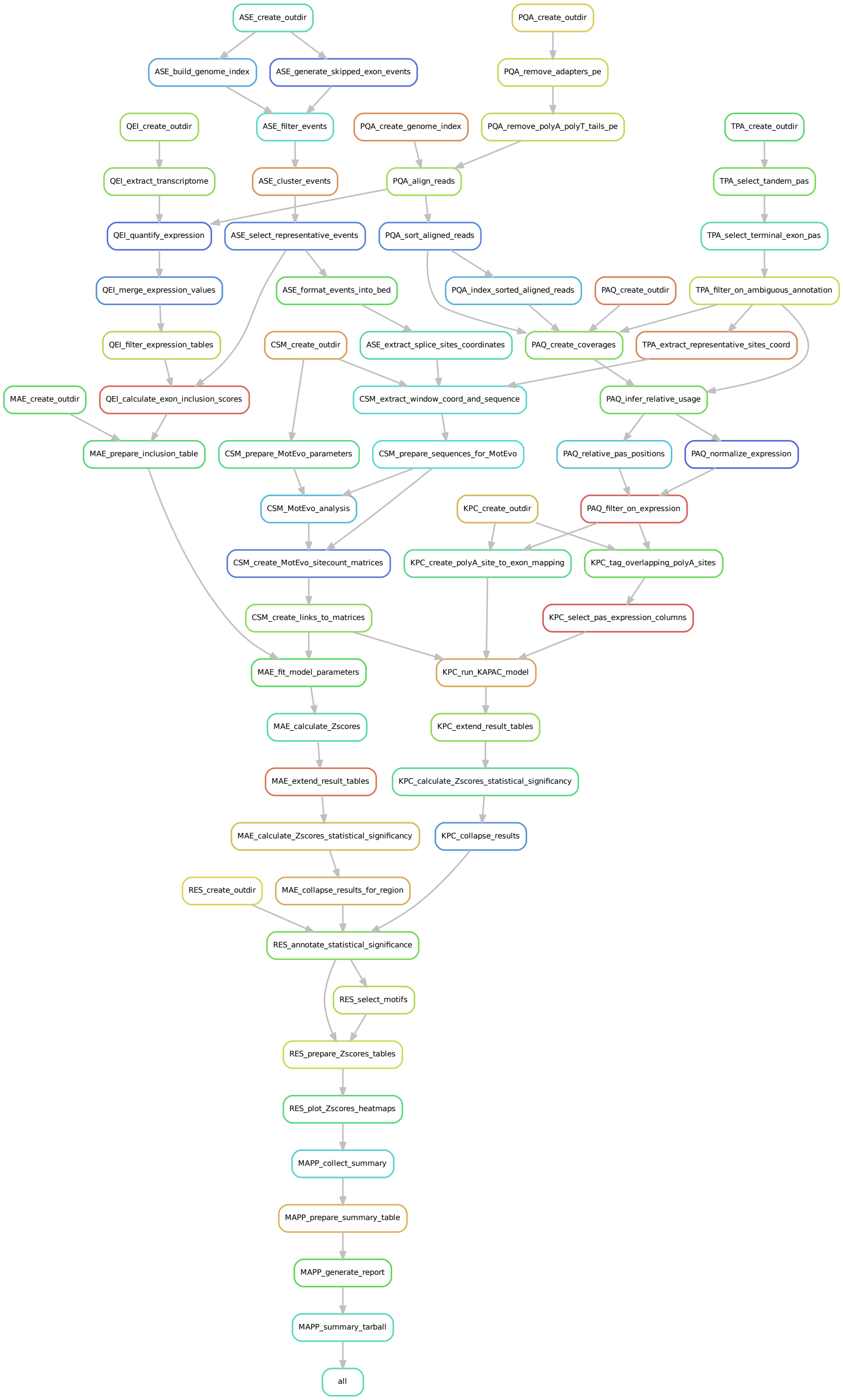

Figure S2. Automatically generated Snakemake rule graph of the MAPP pipeline. Rules of distinct modules of the workflow are prefixed with a three-letter code. Four additional rules are added at the end of the workflow (prefix: *MAPP*) which generate a compressed HTML-formatted report of the MAPP result.

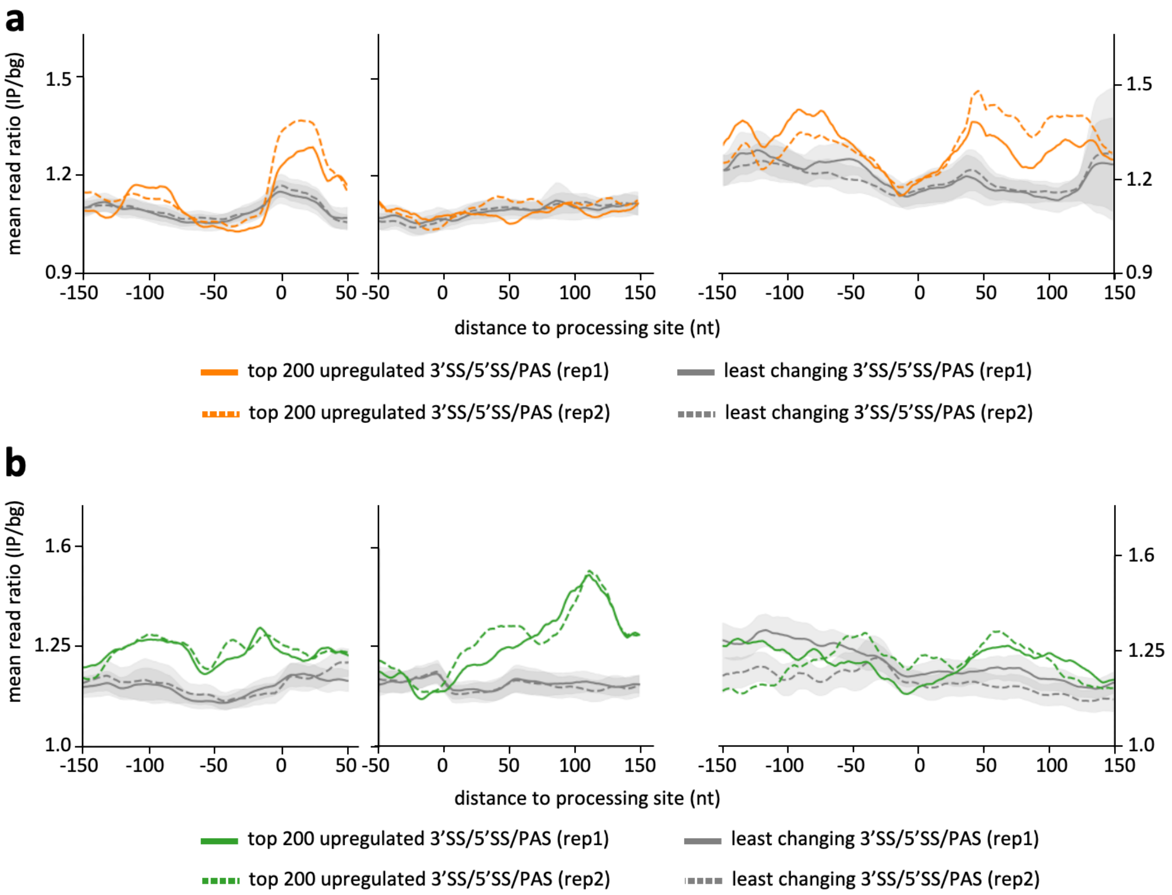

Figure S3. Smoothened (+/- 5 nt) eCLIP-based coverage profiles for distinct RNA-binding proteins generated and presented as in Fig.2 of the main text but in the K562 cell line: **(a)** HNRNPC eCLIP **(b)** RBFOX2 eCLIP.

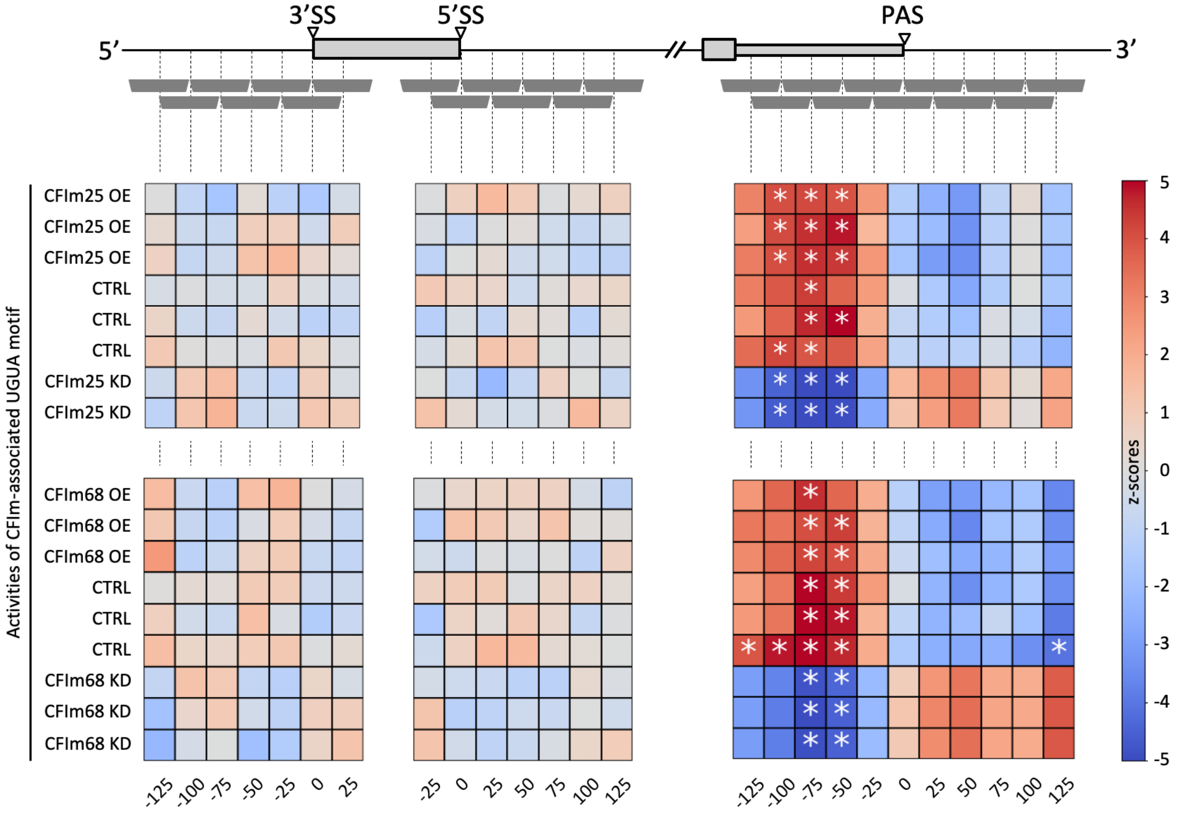

Figure S4. Impact maps of CFIm-associated sequence motif (UGUA) inferred with MAPP on a publicly available dataset of CFIm25 and CFIm68 overexpression and knock-down. Dataset accession number: GSE179630

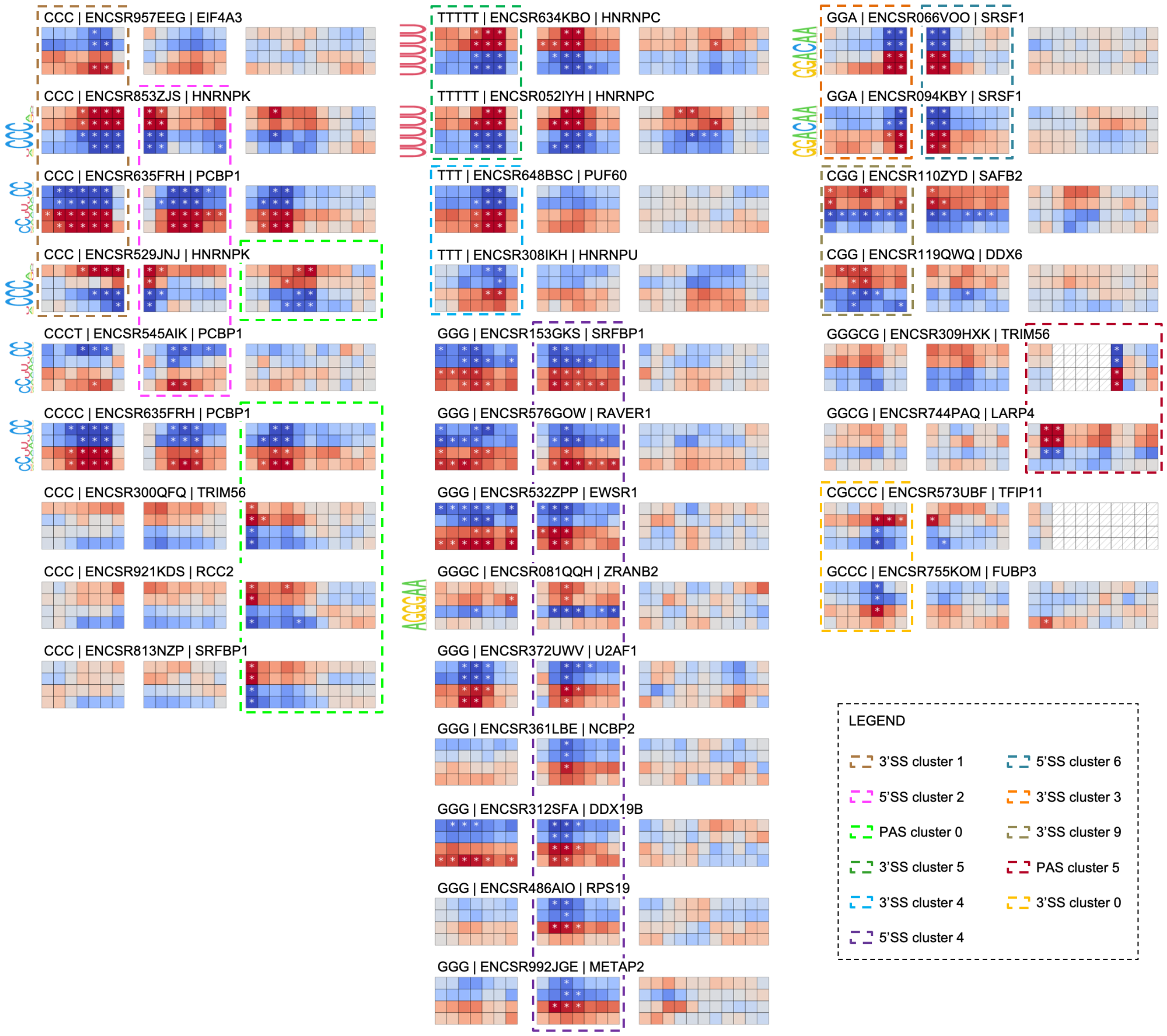

Figure S5. Clustering of the ENCODE project top ranked MAPP results for 5' splice sites (5'SS), 3' splice sites (3'SS) and poly(A) sites (PAS) performed as described in the Supplementary Methods section 2.13. K-mers which cluster together are marked with dashed boxes. Additionally, position weight matrices from the curated ATtRACT database which correspond to the targeted protein are reported next to the impact maps, where available and in decent accordance to the top ranked motif.

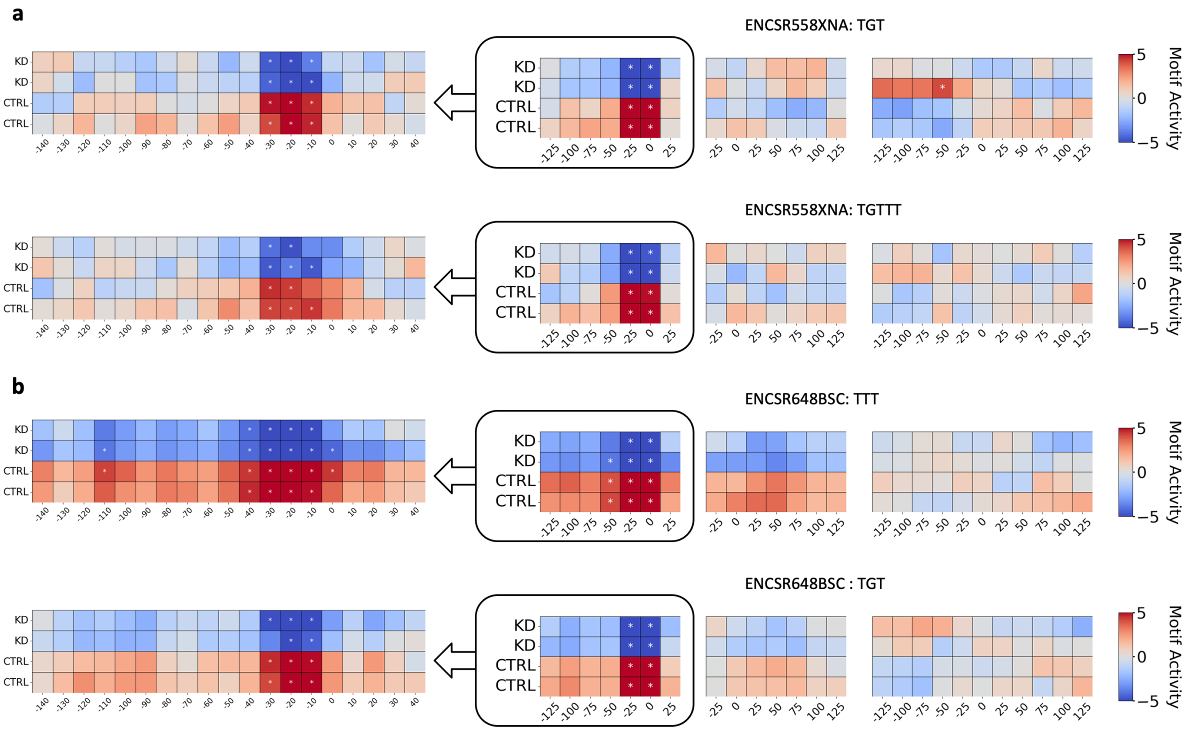

Figure S6. Impact maps of the top two most significant k-mers reported by MAPP after analyzing two PUF60 knock-down experiments from the ENCODE project. (a) PUF60 knock-down in the K5643 cell line and (b) in the HepG2 cell line. Right side: Results based on the windows used by MAPP per default. Left side: In order to obtain more fine-grained insight into the position-specific activity of the RBP we rerun MAPP using more narrow sliding windows around the 3'SS (20nt in length, slided by 10nt).

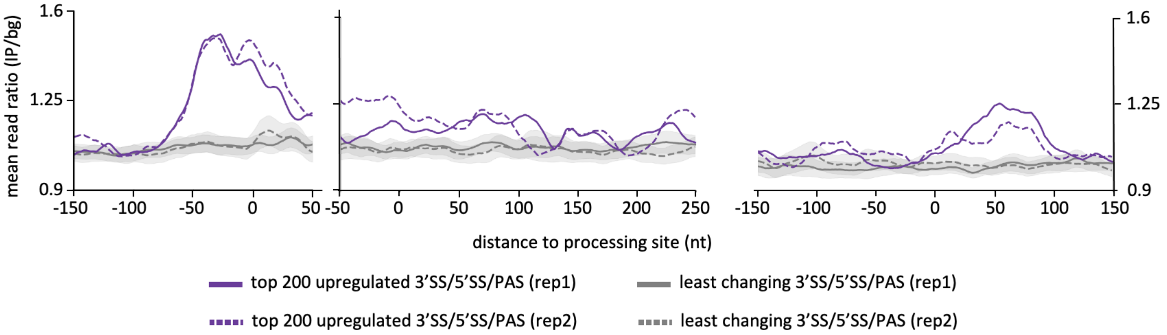

Figure S7. Smoothened (+/- 5 nt) eCLIP-based coverage profiles for PTBP1 RBP generated and presented as in Fig.4 of the main text but in K562 cell line.

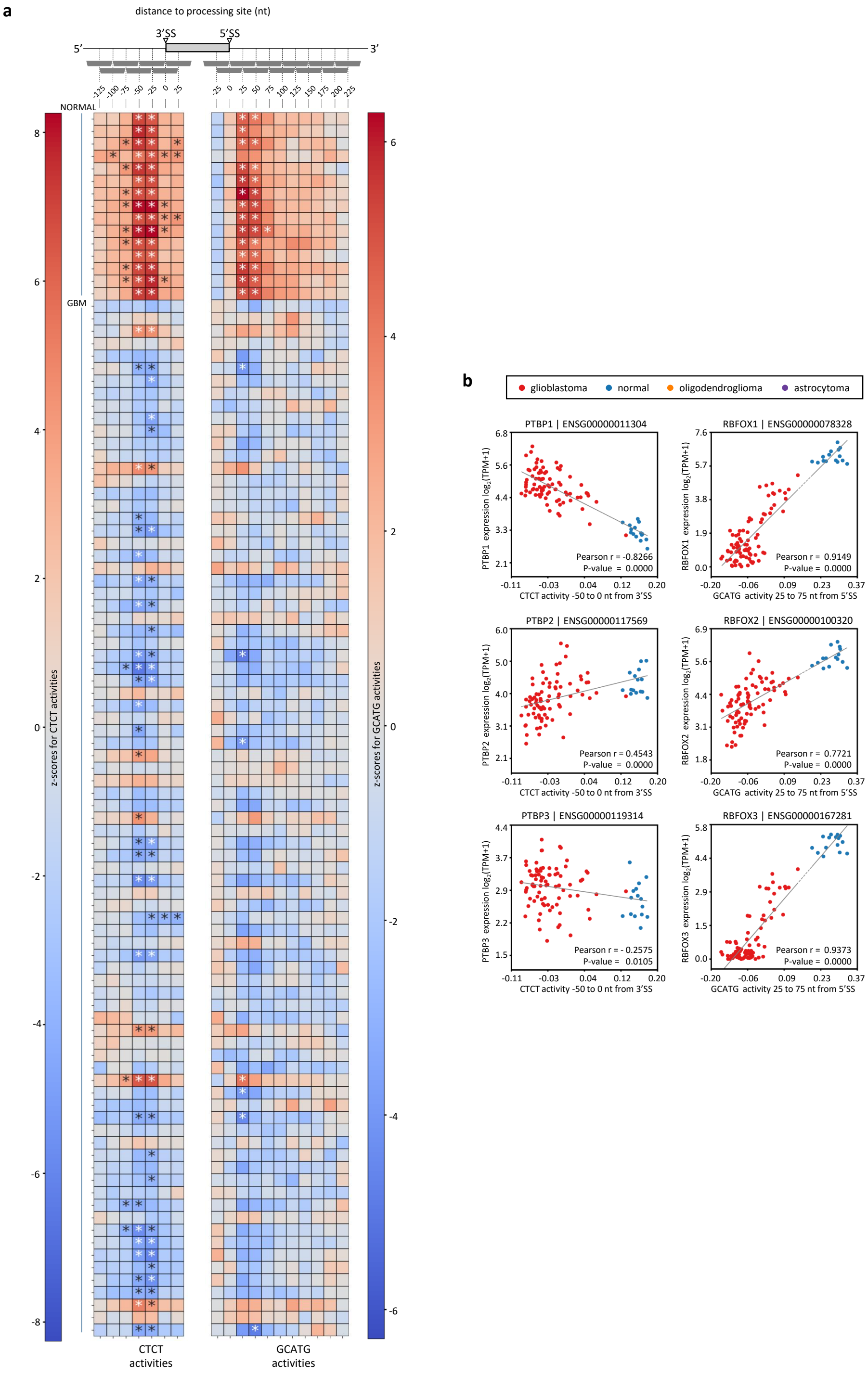

Figure S8. **(a)** MAPP results for normal brain (NORMAL, n=15) and glioblastoma (GBM, n=83) samples (Dataset accession: GSE147352) for the PTBP-bound CTCT k-mer (left panel) as well as for the RBFOX-bound GCATG k-mer (right panel). The exact region definitions for each window are indicated on top of both impact maps. MAPP was run in k-mer mode (covering k-mer lengths 3-5) and without a minimum exon length constraint in order to also account for micro-exons prevalent in neurons. **(b)** Scatter plots of the MAPP inferred activities for the region windows indicated versus the PTBP and RBFOX RBP mRNA expression levels. The dashed gray line shows the linear regression.

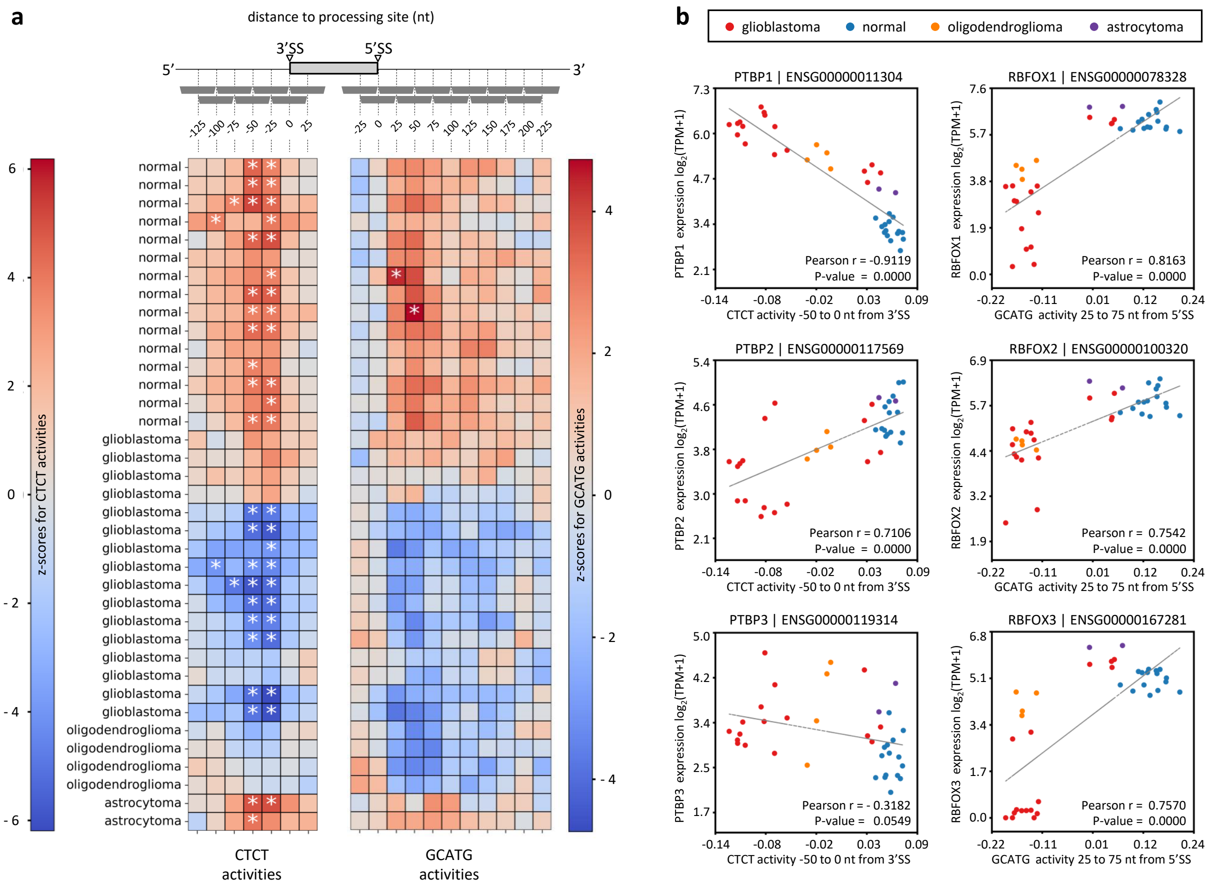

Figure S9. **(a)** MAPP results for normal brain (n=14), glioblastoma (n=16), oligodendroglioma (n=4) and astrocytoma (n=2) samples (Dataset accessions: GSE147352, GSE185861 (two runs for each donor)) for the PTBP-bound CTCT k-mer (left panel) as well as for the RBFOX-bound GCATG k-mer (right panel). Region definitions for the MAPP windows are shown on top of each impact map. MAPP was run in k-mer mode (covering k-mer lengths 3-5). There was no constraint for a minimum exon length in order to also account for micro-exons in neurons. **(b)** Scatter plots of the MAPP inferred activities for the indicated region windows versus the PTBP and RBFOX RBP mRNA expression levels observed for each sample. The dashed gray line shows the linear regression.

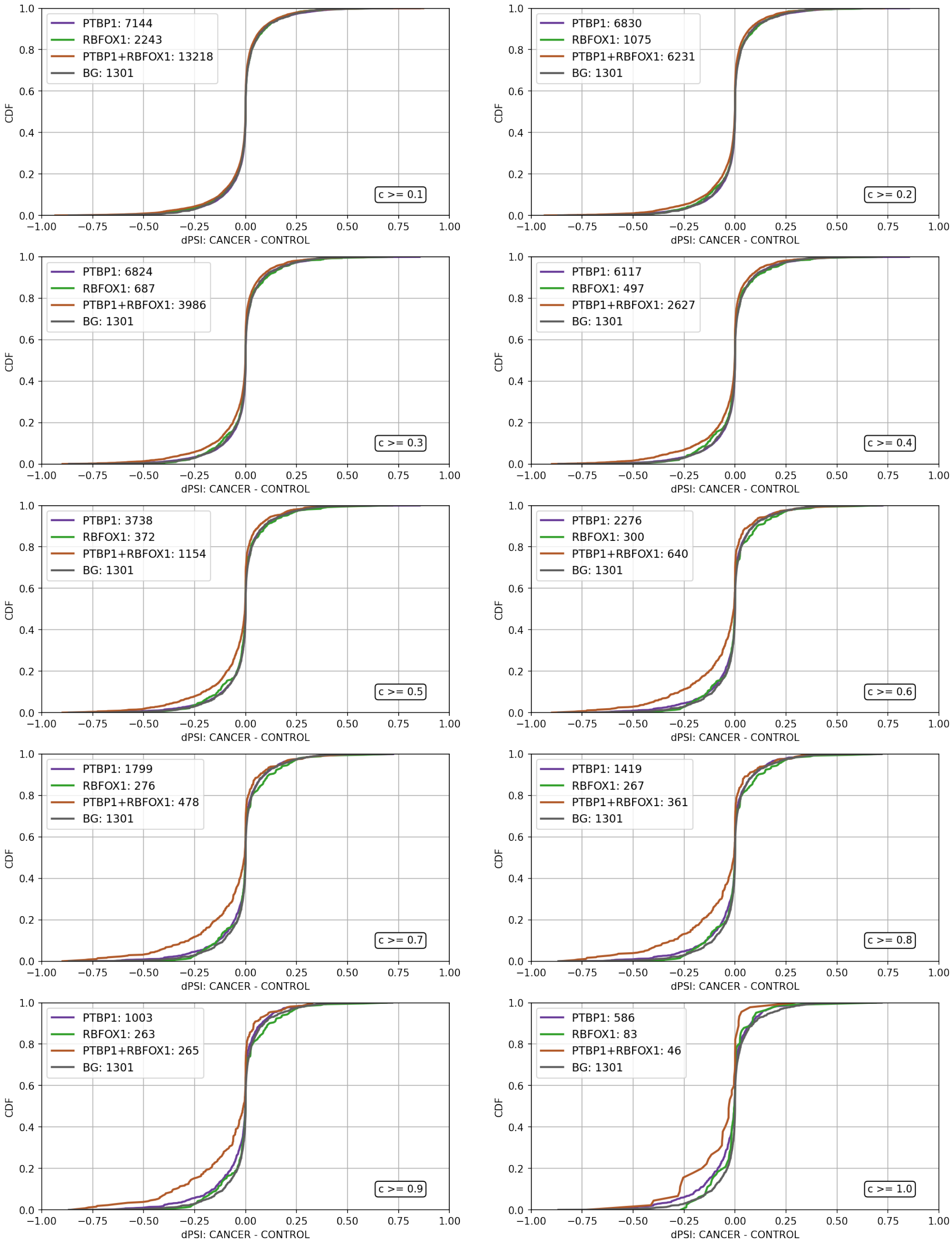

Figure S10. Distribution of differences between percent-spliced-in averages (dPSI) observed in glioblastoma (GBM; n=5) versus normal brain (NORMAL; n=5) for cassette exons regulated by PTBP1 and/or RBFOX according to their binding probabilities within the regions inferred by MAPP to be significantly regulated, using increasing binding probability ($c \geq$) cutoffs. The number of cases is indicated in the legend.

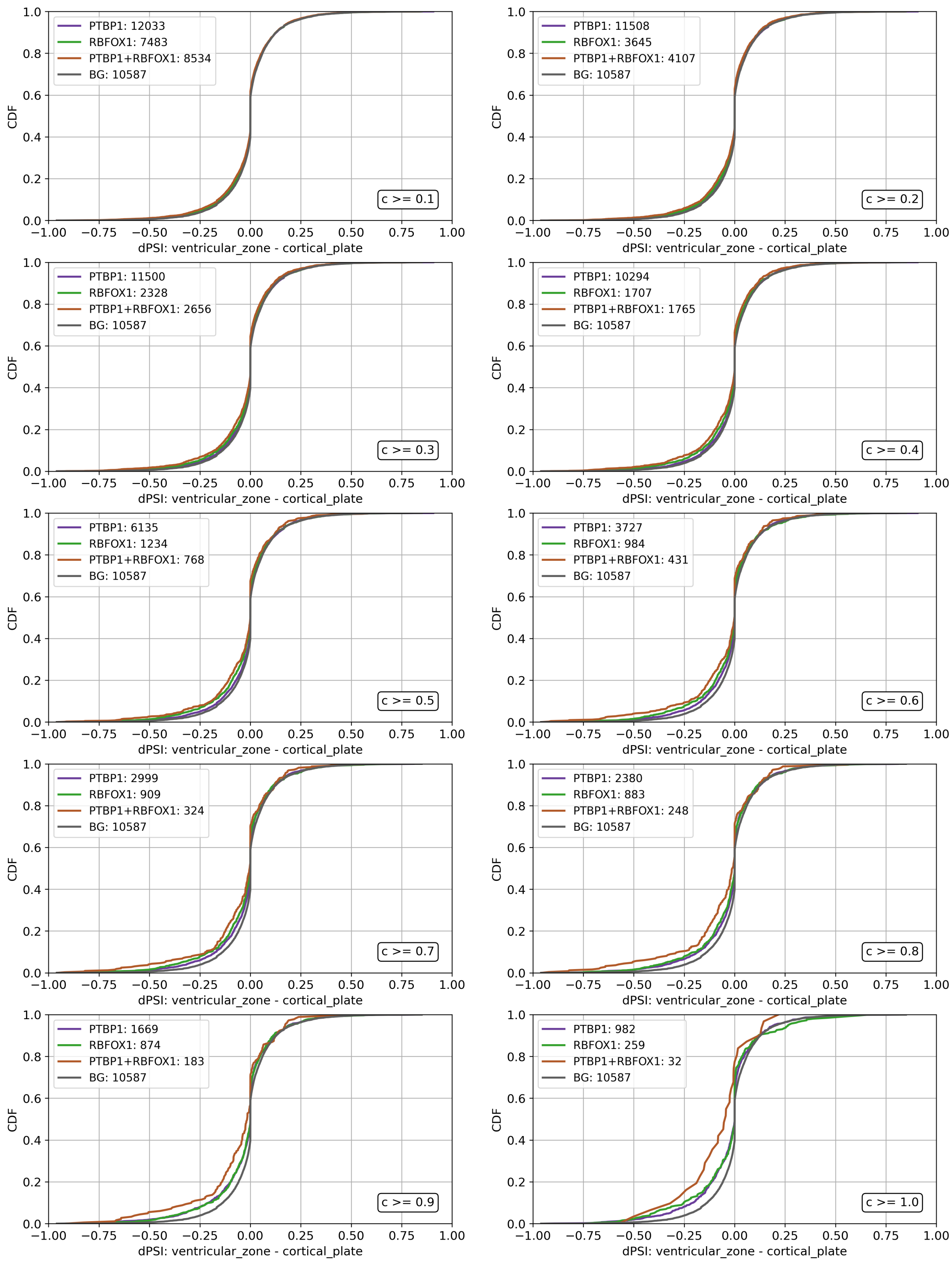

Figure S11. Distribution of differences between percent-spliced-in averages (dPSI) observed in the human fetal neurocortical tissue dataset (accession: GSE38805) for cassette exons regulated by PTBP1 and/or RBFOX according to their binding probabilities within the regions inferred by MAPP to be significantly regulated, using increasing binding probability (c $\geq$) cutoffs. The number of cases is indicated in the legend. Compared conditions involve cortical plate (n=6) and ventricular zone (n=6).

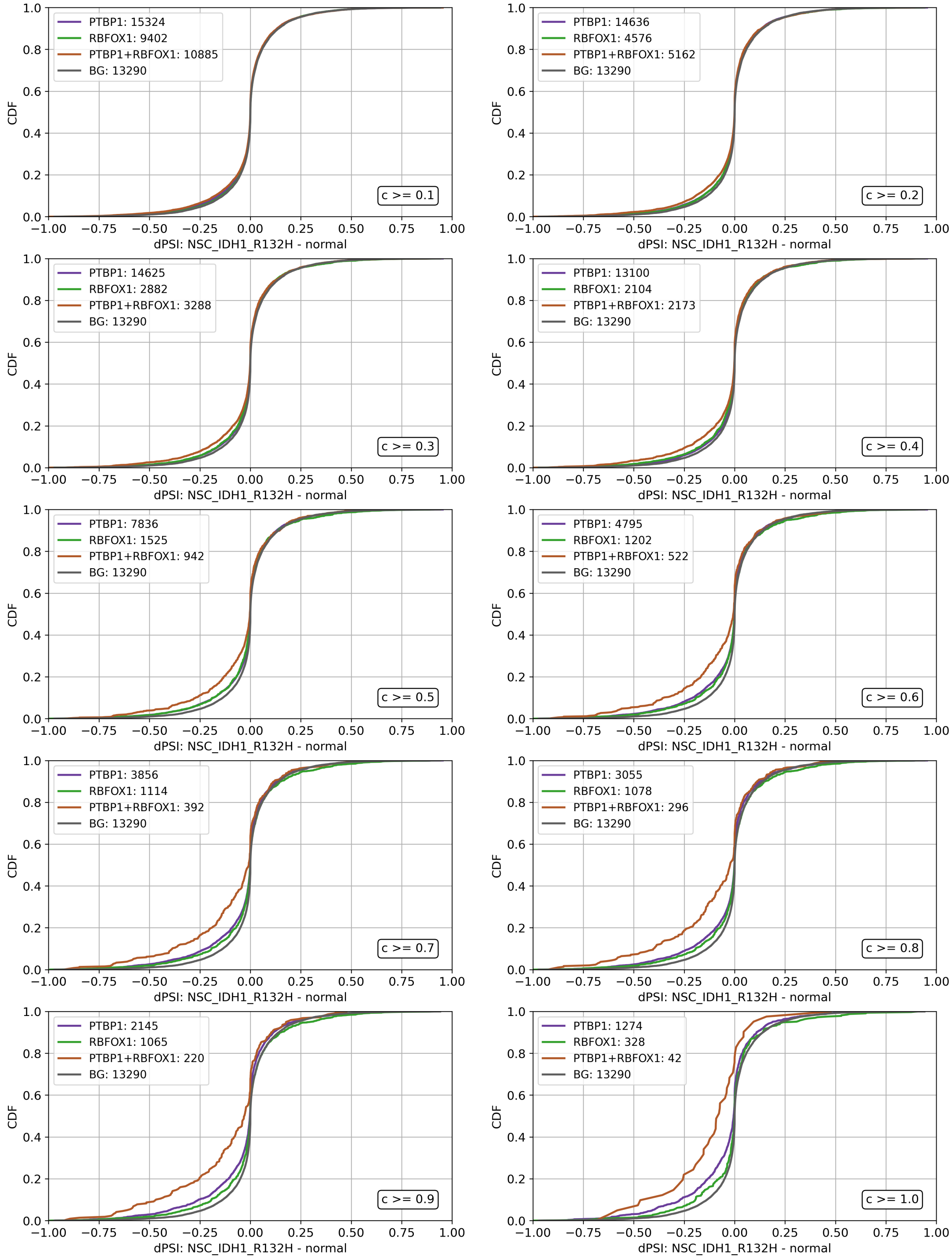

Figure S12. Distribution of differences between percent-spliced-in averages (dPSI) observed in normal brain tissue (n=9) versus a neural stem cell model (n=9) for cassette exons regulated by PTBP1 and/or RBFOX according to their binding probabilities within the regions inferred by MAPP to be significantly regulated, using increasing binding probability (c $\geq$) cutoffs. The number of cases is indicated in the legend. Data accession numbers: PRJNA798408, GSE147352.

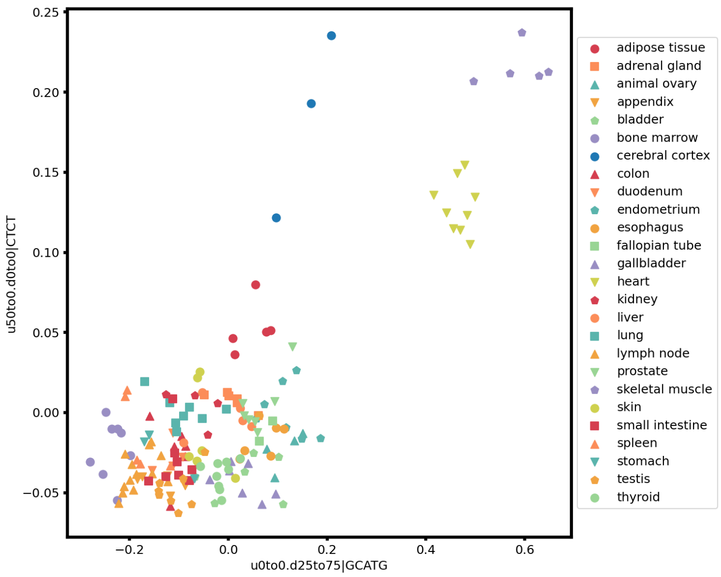

Figure S13. Inferred activities of the CTCT and GCATG k-mers on 3' splice site (3'SS) and 5' splice site (5'SS) processing, respectively, in the indicated windows relative to the splice sites, i.e. -50 nt to 0 nt upstream of 3'SS and +25nt to +75nt downstream of 5'SS, in distinct human tissues listed in the legend. Dataset accession: PRJEB4337, PRJEB6971. Only unstranded samples were considered. All analysed samples are listed in Supplementary Table S1.

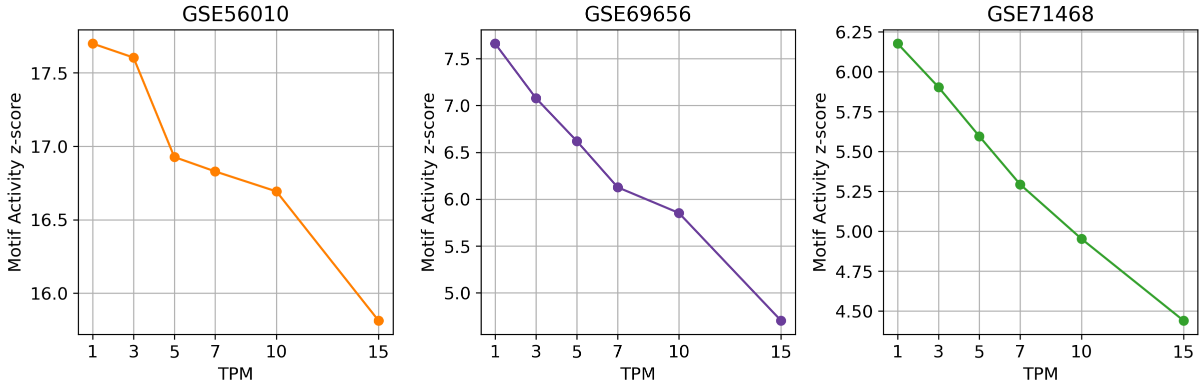

Figure S14. MAPP reported motif activity z-scores of the top ranked motifs acting around its respective sites as a function of minimum TPM cutoff for expressed genes to be considered in MAEI: **(a)** HNRNPC knock-down; motif TTTTT acting on 3'SS, **(b)** PTBP1/2 knock-down; motif TCT acting on 3'SS , and **(c)** RBFOX1 overexpression; motif GCATG acting on 5'SS.

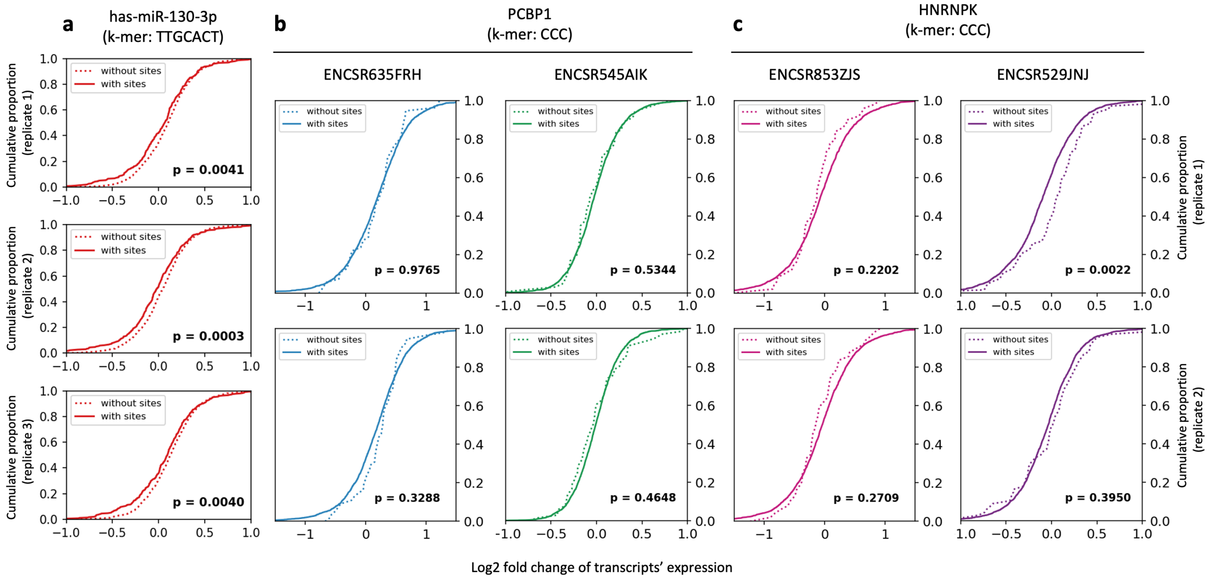

Figure S15. Cumulative distributions of transcripts' expression log2 fold changes for transcripts which contain specific k-mers in their 3'UTR sequences and those with zero counts. Samples' replicates are stacked vertically. Five distinct experiments are presented: **(a)** hsa-miR-130-3p overexpression, **(b)** two knock-down experiments of PCBP1 from the ENCODE dataset, and **(c)** two knock-down experiments of HNRNPK from the ENCODE dataset.

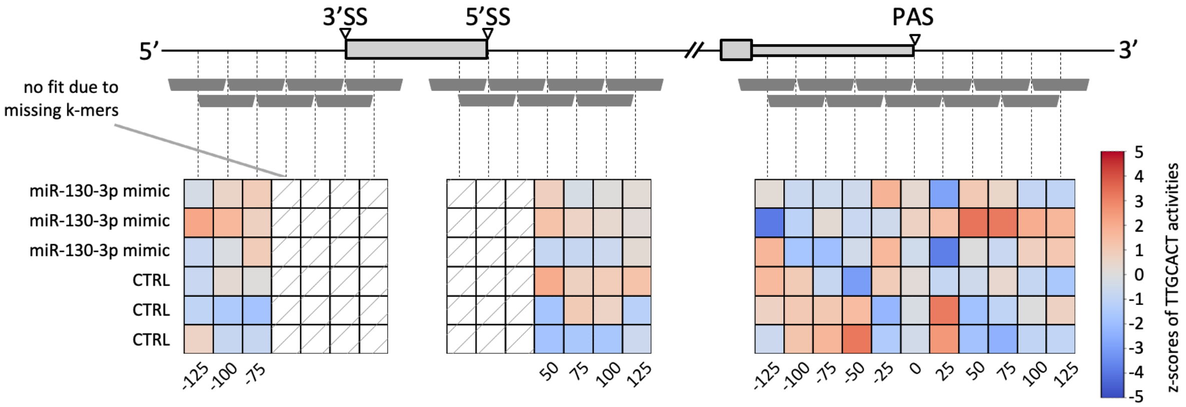

Figure S16. Impact map for the miR-130-3p binding sequence (TTGCACT) inferred with MAPP from the samples of a miR-130-3p overexpression dataset (Accession Number: GSE204705).

2 Supplementary Methods

The main parameters relevant for a MAPP run are specified within the MAPP config file. Detailed information on how to create the config file and adapt its parameters are provided in the MAPP code

repository. The individual MAPP modules, including the corresponding config file parameters, are described in subsections 2.1 to 2.10 below.

2.1 Data preprocessing and quality analysis

Snakemake rules which belong to this module are marked with a three-letter name space: *PQA*.
The MAPP config file parameters relevant for the PQA module are shown in Figure S17.

2.1.1 PQA Module details

Relevant information about the RNA-Seq samples that should be analysed by MAPP are provided by an TSV-formatted design table file, which is provided to the MAPP pipeline via the *analysis_design_table* config file parameter. More information on the design table's structure is provided in the codebase's README file. Processing of raw sequencing reads in FASTQ format starts with two consecutive runs of cutadapt tool [1]. First, the sequences of adapters are removed and subsequently we trim poly(A) tails. Cutadapt parameters were set to: *-e 0.1*, *–pair-filter any*, *–times 1*, *–trim-n*, *–mininum-length 10* and additionally for the tail trimming: *-O 1*. Following that the reads are mapped to both genome and transcriptome using STAR aligner [2], which also - based on the provided resources provided with configuration flags: *genomic_sequence* and *genomic_annotation*) - builds a genomic index prior aligning the reads. Genomic index should be always constructed with a specific read length in mind, thus the STAR parameter *sjdbOverhang* may be also specified in the configuration file for MAPP. In case the genome had already been indexed the user may provide it through the *genomic_index* field and skip this step in the workflow. Parameter *storage_efficient* denotes whether the trimmed reads should be decompressed prior to aligning them to the genome or used directly as STAR's input. Aligner's-specific command-line parameters were set to: *–twopassMode Basic*, *–outSAMunmapped None*, *–outSAMattributes All*, *–outReadsUnmapped None*, *–outFilterType BySJout*, *–alignEndsType Local*, *–outFilterMismatchNoverLmax 0.1*, *–outFilterScoreMinOverLread 0.66*, *–outFilterMatchNminOverLread 0.66*, *–outFilterMultimapNmax 10*, *–outFilterMultimapScoreRange 0*, *–readFilesCommand zcat*, *–outSAMtype BAM Unsorted*, *–quantMode TranscriptomeSAM*. Obtained alignments in BAM format were then sorted and indexed with samtools [3].

```
genomic_sequence: ""
genomic_annotation: ""
genomic_index: ""
analysis_design_table: ""
sjdbOverhang: 100
transcript_biotypes: "protein_coding"
quality_check: True
min_median_TIN_score: 50.0
RNASeQC_min_mapping_rate: 0.95
RNASeQC_min_unique_rate_of_mapped: 0.90
RNASeQC_min_high_quality_rate: 0.85
RNASeQC_max_intergenic_rate: 0.1
RNASeQC_max_rRNA_rate: 0.01
storage_efficient: False
```

Figure S17. MAPP config file parameters for the PQA module

If the *quality_check* parameter is set MAPP will also carry out quality control of the data and, prior to the downstream analysis, filter-out such RNA-Seq samples which do not meet specified criteria. For the quality analysis we use metrics provided by RNA-SeQC [4] and TIN score calculated as in RSeQC package [5]. According to RNA-SeQC's manual, we filter RNA-Seq samples on the following parameters: minimal fraction of mapped reads (*RNASeQC_min_mapping_rate*), minimal fraction of unique reads mapped (*RNASeQC_min_unique_rate_of_mapped*), minimal fraction of high quality reads

(*RNASeQC_min_high_quality_rate*), maximum fraction of intergenic reads (*RNASeQC_max_intergenic_rate*) and maximum fraction of rRNA-mapped reads (*RNASeQC_max_rRNA_rate*), for which the default values are specified in the supplementary figure above. As for the TIN score, parameter (*min_median_TIN*) denotes the median-based filter calculated over the set of all transcripts in the genomic annotation of a given biotype (*transcript_biotype* field in the configuration file). The following command line flags for the TIN score calculation script were specified: *-c 0*, *-n 100*. Additionally all samples are handled over to FastQC [6] which generates additional per-sample summary report (FastQC parameters: *–format fastq*, *–nogroup*, *–extract*, *–kmers 7*).

2.1.2 PQA Module software tools and scripts workflow

In the MAPP code repository the PQA module is located under: *modules/PREPROCESSING*.
The workflow consist of the following rules (see Figure S18):

- **PQA_create_outdir** : creates top-level output directory to store all module results.
- **PQA_prepare_adapters_textfiles** : refines the list of potential RNA-sequencing adapters with those specified in the design table; executed only if the quality filter flag is turned on.
- **PQA_run_FastQC** : runs the FastQC tool for quality control of the input sequencing data, executed only if the quality filter flag is turned on.
- **PQA_create_genome_index** : prepares an index for the input genomic sequence which will be used later during the read mapping phase.
- **PQA_remove_adapters_se** : executes cutadapt tool to remove sequencing adapters from the 3'ends of the reads; this rule is dedicated to single-end sequencing data.
- **PQA_remove_adapters_pe** : executes cutadapt tool to remove sequencing adapters from the 3'ends of the reads; this rule is dedicated to paired-end sequencing data.
- **PQA_remove_polyA_polyT_tails_se** : executes cutadapt tool to remove poly(A) or poly(T) tails from the 3'ends of the reads; this rule is dedicated to single-end sequencing data.
- **PQA_remove_polyA_polyT_tails_pe** : executes cutadapt tool to remove poly(A) or poly(T) tails from the 3'ends of the reads; this rule is dedicated to paired-end sequencing data.
- **PQA_align_reads** : executes STAR tool in order to align trimmed sequencing reads against both genome and transcriptome.
- **PQA_sort_aligned_reads** : uses samtools to sort the alignment files (sequencing reads aligned against the genome).
- **PQA_index_sorted_aligned_reads** : uses samtools to index the sorted alignment files.
- **PQA_collapse_genomic_annotation** : collapses isoforms into single transcript per gene based on the GTF-formatted annotation and remove overlapping intervals between genes.
- **PQA_mapping_quality_analysis** : runs RNA-SeQC tool on each sample to calculate various quality metrics on the aligned sequencing reads.
- **PQA_merge_mapping_quality_tables** : merges per-sample RNA-SeQC quality metrics into a TSV-formatted table.
- **PQA_extract_transcripts_as_bed12** : extracts all protein-coding transcript from the genomic annotation and save them in a bed12 format.

- **PQA_calculate_TIN_scores** : assess RNA degradation rate by calculating per-sample TIN score.
- **PQA_merge_TIN_scores** : merges per-sample TIN scores into a TSV-formatted table.
- **PQA_calculate_median_TIN_score** : calculates per-sample median TIN score over the set of all protein-coding transcripts.
- **PQA_filter_design_table** : filters sequencing samples from the design table based on the previously calculated scores: median TIN score and various RNA-SeQC metrics (described in the previous sub-section).
- **PQA_all** : final rule of the workflow which gathers the output of all previous processing steps.

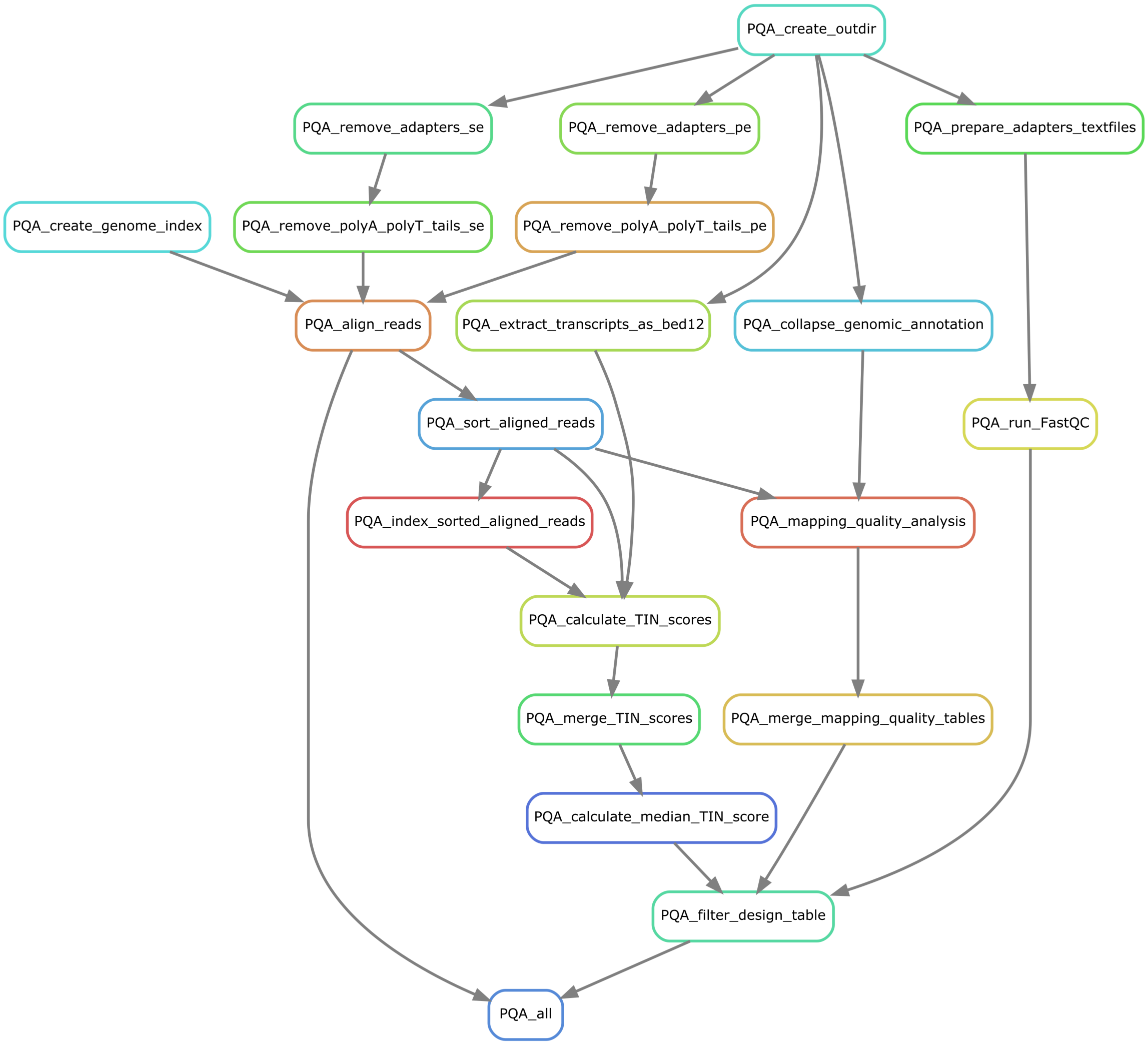

Figure S18. Snakemake rulegraph for the PQA module of MAPP.

2.2 Selection of cassette exons

Snakemake rules which belong to this module are marked with a three-letter namespace: *ASE*.
The MAPP config file parameters relevant for the ASE module are presented in Figure S19.

2.2.1 ASE Module details

We select a set of cassette (also known as 'skipped') exons based solely on the standard ENSEMBL genomic annotation, version: hg38 (genomic resources provided as *genomic_sequence* and *genomic_annotation* configfile fields). We first run SUPPA2 [7] to generate all skipped exon events and then filter them for minimal length sufficient for the downstream analyses (with the *min_exon_len* and *max_exon_len* configfile fields). We focus only on records annotated as *protein_coding*, which was set by the *transcript_biotype* field, also in the configuration file. Obtained events are further clustered according to a mutual coverage dissimilarity measure:

$$d(e_1, e_2) = 1 - min(\frac{len(e_1 \cap e_2)}{len(e_1)}, \frac{len(e_1 \cap e_2)}{len(e_2)}) \quad (1)$$

Where e_1 and e_2 denote two exons and with $\cap$ we take only the overlap between then. We applied a hierarchical clustering with a maximum linkage strategy and set 0.05 as a cluster linkage cutoff parameter (config parameter: *exon_clustering_max_coverage_distance*).

```
genomic_sequence: ""
genomic_annotation: ""
min_exon_len: 100
max_exon_len: 1000000
transcript_biotypes: "protein_coding"
exon_clustering_max_coverage_distance: 0.05
```

Figure S19. MAPP config file parameters for the ASE module

From every cluster we selected one event with the highest number of distinct transcripts that support it. Such an event we call a representative exon. For all representative exons we keep the information which transcripts of a given gene include it as well as the list of all transcripts for a given gene (both provided by SUPPA2). We also save the coordinates of 3' and 5' splice-sites of these representatives.

2.2.2 ASE Module software tools and scripts workflow

In the MAPP code repository the ASE module is located under: *modules/EXTRACT_AS_EXONS*.
The workflow consist of the following rules (see Figure S20):

- **ASE_create_outdir** : creates top-level output directory to store all module results.
- **ASE_build_genome_index** : runs samtools to generate an index for the genomic sequence provided in the fasta format.
- **ASE_generate_skipped_exon_events** : executes SUPPA2 tool to generate a set of all cassette exons based on the provided genomic annotation.
- **ASE_filter_events** : filters all cassette exon splicing events based on their minimal/maximal length transcripts' biotype and distance to the chromosomes' ends.
- **ASE_cluster_events** : clusters together largely overlapping cassette exon events.
- **ASE_select_representative_events** : from each of the clusters annotated above selects one representative cassette exon event for further processing.

- **ASE_format_events_into_bed** : saves all selected representative events into a bed format.
- **ASE_extract_splice_sites_coordinates** : from each representative cassette exon extracts the coordinates of its 3'SS and 5'SS and save them into separate bed-formatted files.
- **ASE_all** : final rule of the workflow which gathers the output of all previous processing steps.

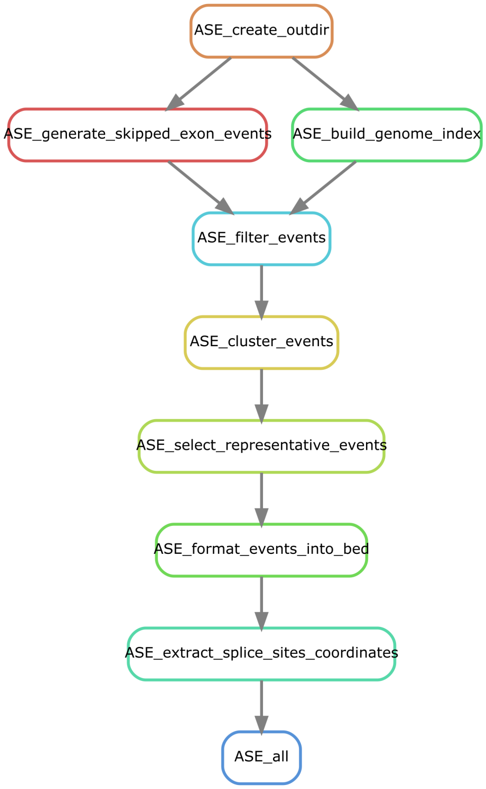

Figure S20. Snakemake rulegraph for the ASE module of MAPP.

2.3 Extraction of tandem poly(A) sites

Snakemake rules which belong to this module are marked with a three-letter namespace: *TPA*. The MAPP config file parameters relevant for the TPA module are presented in Figure S21.

2.3.1 TPA Module details

We select a set of poly(A) sites which may be classified as proximal/distal within a given terminal exon based on a provided BED-formatted poly(A) site atlas as well as a GTF-formatted genomic annotation. These resources are provided in the configuration file by: *PAS_atlas* and *genomic_annotation* fields. Throughout the following study we use PolyASite 2.0 [8] and ENSEMBL annotation, version hg38.

```
PAS_atlas: ""
genomic_annotation: ""
library_type: ""
polyA_sites_min_no_protocol: 1
transcript_biotypes: "protein_coding"
three_prime_end_offset: 0
transcript_locus_offset: 100
tandem_polyA_exon_extension: 200
```

Figure S21. MAPP config file parameters for the TPA module

We focus on all poly(A) sites supported by at least one experimental protocol (atlas-specific information and *polyA_sites_min_no_protocol* config field) but filter for such which are located only on *protein-coding* transcripts (*transcript_biotypes* parameter). We also discard all sites which could be ambiguously annotated to distinct genes. We export coordinates of the resulting tandem polyA sites into a BED-formatted list. Additional configuration parameters for this module include: *three_prime_offset* (downstream extension after the terminal exon end to search in polyA sites) *transcript_locus_offset* (upstream and downstream extension of transcripts' coordinates in search of polyA sites) and *tandem_polyA_exon_extension* (downstream extension for mapping exons with tandem polyA sites to genes).

2.3.2 TPA Module software tools and scripts workflow

In the MAPP code repository the TPA module is located under: *modules/PREPARE_TANDEM_PAS*. The workflow consist of the following rules (see Figure S22):

- **TPA_create_outdir** : creates top-level output directory to store all module results.
- **TPA_select_tandem_pas** : selects only such poly(A) sites from the input annotation which could be categorised as proximal/distal.
- **TPA_select_terminal_exon_pas** : filters tandem poly(A) sites to keep only those which are located on terminal exons.
- **TPA_filter_on_ambiguous_annotation** : intersects the set of tandem poly(A) sites of terminal exons with genomic annotation in the GTF format; retain only such exons which can be associated to a single gene unambiguously.
- **TPA_extract_representative_sites_coord** : extracts a representative coordinate for each of the tandem poly(A) sites and save them in the bed format.
- **TPA_all** : final rule of the workflow which gathers the output of all previous processing steps.

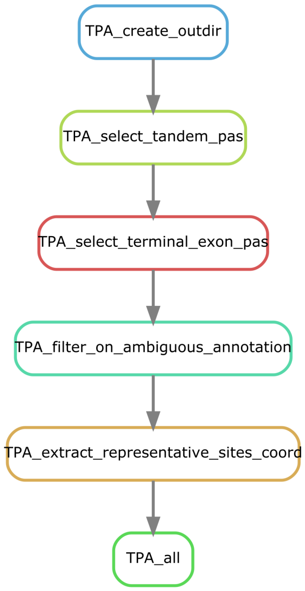

Figure S22. Snakemake rulegraph for the TPA module of MAPP.

2.4 Quantification of exon inclusion

Snakemake rules which belong to this module are marked with a three-letter namespace: *QEI*. The MAPP config file parameters relevant for the QEI module are presented in Figure S23.

2.4.1 QEI Module details

In order to quantify transcripts' expression based on transcriptomic alignments we run Salmon [9]. Having obtained per-sample, per-transcript TPM-normalized expression values we use the previous information to collapse the scores into a more exon-centric level: for each representative exon (section 2.2) in every sample we calculate two values: summed up total TPM expression of all transcripts which include this exon ($i_{e,s}$) as well as summed up total TPM expression of all transcripts of a given gene ($t_{e,s}$). Configfile parameters utilised in this module comprise of previously described: *genomic_sequence*, *genomic_annotation* and *analysis_design_table*.

```
genomic_sequence: ""
genomic_annotation: ""
analysis_design_table: ""
```

Figure S23. MAPP config file parameters for the QEI module

2.4.2 QEI Module software tools and scripts workflow

In the MAPP code repository the TPA module is located under: *modules/QUANTIFICATION*. The workflow consist of the following rules (see Figure S24):

- **QEI_create_outdir** : creates top-level output directory to store all module results.
- **QEI_extract_transcriptome** : runs cufflinks tool in order to generate transcriptome based on a given genomic sequence and annotation resources.

- **QEI_quantify_expression** : executes salmon tool to quantify per-sample expression of genes and transcripts.
- **QEI_merge_expression_values** : merges per-sample expression scores represented as TPM values into a TSV-formatted table.
- **QEI_filter_expression_tables** : subsets only these RNA-Seq samples which passed the quality control filters (applicable to MAPP runs for which quality-based filtering was turned on).
- **QEI_calculate_exon_inclusion_scores** : calculates the inclusion fractions for all representative cassette exons and represents them as total expression of all transcripts which: (1) include, (2) could possibly include them.
- **QEI_all** : final rule of the workflow which gathers the output of all previous processing steps.

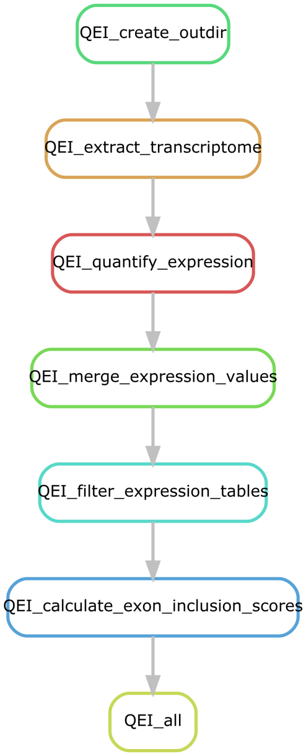

Figure S24. Snakemake rulegraph for the QEI module of MAPP.

2.5 Quantification of poly(A) sites' expression

Snakemake rules which belong to this module are marked with a three-letter namespace: *PAQ*. The MAPP config file parameters relevant for the PAQ module are presented in Figure S25.

2.5.1 PAQ Module details

In order to quantify the expression of distinct tandem poly(A) sites we employed our previously developed tool: PAQR [10]. The method takes as input genomic alignments of RNA-Seq reads in BAM format and a BED-formatted list of poly(A) sites of interest. It infers expression of distinct sites directly from the coverage profiles in their proximity (please see original publication for more details).

```
analysis_design_table: ""
library_type: ""
coverage_extension: 200
cvg_start2prox_minDist: 250
readLength: 100
relUse_minLength_meanCvg: 100
relUse_minMeanCvg_perSample: 5
relUse_distal_ds: 200
relUse_distal_ds_maxCvg: 10
relUse_min_cluster_distance: 200
relUse_us_reg_for_best_breakPoint: 200
relUse_mse_ratio_threshold: 0.5
pas_expression_pseudocount: 1
```

Figure S25. MAPP config file parameters for the PAQ module

PAQR may be parametrized with the following configuration file parameters: *analysis_design_table* (as in previous modules), *library_type* (either: stranded or unstranded), *coverage_extension* (length of a downstream extension of the coverage profile following an exon end), *cvg_start2prox_minDist* (minimum distance between the exon start and the most proximal poly(A) site), *relUse_minLength_meanCvg* (minimum length of the region that needs to be available to calculate a mean coverage and the mean squared error), *relUse_minMeanCvg_perSample* (per-sample minimum mean coverage required for an exon to be considered in the analysis), *readLength* (as in the RNA-seq library), *relUse_distal_ds* (length of the region downstream of the exon, necessary to search for zero coverage downstream of the distal site), *relUse_distal_ds_maxCvg* (maximum percentage of the coverage at the exon start which is allowed for the mean coverage in the downstream region of a valid distal site), *relUse_min_cluster_distance* (distance until which PAS clusters are merged), *relUse_us_reg_for_best_breakPoint* (upstream extension added to the PAS cluster's end during the check for global break points), *relUse_mse_ratio_threshold* (upper limit on the ratio of coverage mean squared error values after and before including a new break point), *pas_expression_pseudocount* (TPM pseudocount to be added in the final expression table). As an output it provides TPM-normalized expression of poly(A) sites in all samples and, additionally, a list of their relative position within respective terminal exons. Furthermore, we filter the output table to keep tandem sites only located on such exons for which all their sites are considered as expressed in all analyzed samples.

2.5.2 PAQ Module software tools and scripts workflow

In the MAPP code repository the PAQ module is located under: *modules/PAQR*.
The workflow consist of the following rules (see Supplementary Fig. S26):

- **PAQ_create_outdir** : creates top-level output directory to store all module results.
- **PAQ_create_coverages** : extracts read coverages of tandem poly(A) sites from the genomic alignment files.
- **PAQ_infer_relative_usage** : infers expression and usage of distinct tandem poly(A) sites bases on read coverage profiles.

- **PAQ_normalize_expression** : normalizes the expression of poly(A) sites by the number of mapped reads to obtain the TPM values.
- **PAQ_relative_pas_positions** : obtains relative positions of the poly(A) sites within their respective terminal exons.
- **PAQ_filter_on_expression** : removes all such terminal exons for which any site was not expressed in all of the samples.
- **PAQ_all** : final rule of the workflow which gathers the output of all previous processing steps.

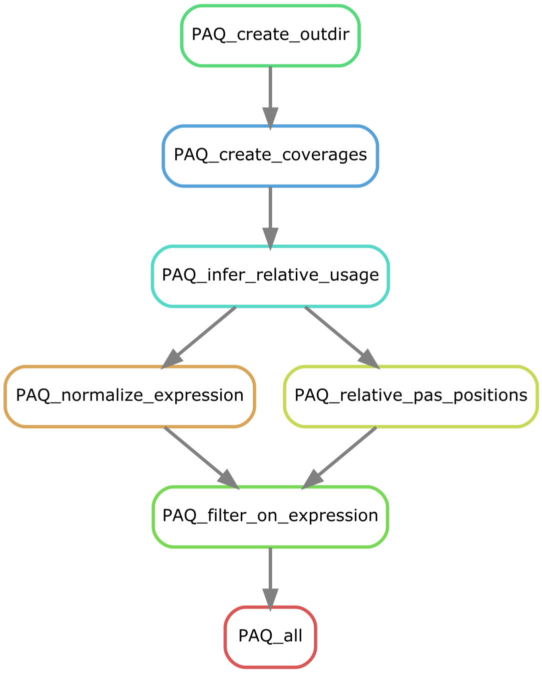

Figure S26. Snakemake rulegraph for the PAQ module of MAPP.

2.6 Sitecount Matrices

Snakemake rules which belong to this module are marked with a three-letter namespace: *CSM*. The MAPP config file parameters relevant for the CSM module are presented in Figure S27.

2.6.1 CSM Module details

Both statistical models for discovering regulators of cassette exon inclusion and poly(A) sites' usage require tables containing quantified information about binding sites of distinct RNA-binding proteins within a certain distance relative to the site of interest. We call such tables 'sitecount matrices'. In case of alternative splicing analysis we define a range around 3'SS and 5'SS. For the alternative polyadenylation analysis we focus around tandem poly(A) sites. We employ a 'sliding-window' strategy in order to gain a better resolution into the positional-dependent effect of RBPs binding sites on the mRNA maturation process. Thus, the pipeline generates multiple sitecount matrices for 3'SS, 5'SS and poly(A) sites - each one corresponding to a unique window defined relatively to the site of interest.

```
genomic_sequence: ""
window_size: 50
matrix_type: "kmers"
3ss_region_up: 150
3ss_region_down: 50
5ss_region_up: 50
5ss_region_down: 150
pas_region_up: 150
pas_region_down: 150
k_min: 3
k_max: 6
MotEvo_bg_binding_prior: 0.99
MotEvo_min_binding_posterior: 0.01
MotEvo_Markov_chain_order: 1
PWM_directory: ""
seqlogo_directory: ""
```

Figure S27. MAPP config file parameters for the CSM module

MAPP pipeline can be run in two modes: *kmer-* and *pwm-based* (configfile parameter: *matrix_type*), depending on how the user chooses to generate sitecount matrices. In both cases the module reads in BED-formatted files with the positions of 3'SS and 5'SS of cassette exons as well as locations of tandem poly(A) sites (all previously generated) and produces files with coordinates of distinct windows around them. Window length is specified in the config field: *window_size*), relative window endpoints are provided by: *3ss_region_up*, *3ss_region_down*, *5ss_region_up*, *5ss_region_down*, *pas_region_up*, *pas_region_down*. We extract genomic sequences of the regions encoded in these windows with Pybedtools [11] (genomic sequence provided by the *genomic_sequence* parameter). In the former mode we sum up the occurrences of distinct k-mers over the sequence of the whole window (making sure not to overcount short overlapping homomeric subsequences) and the resulting sitecount matrix contains raw counts. Range of the k-mer's length is given by the configfile fields: *k_min* and *k_max*. In the *pwm-based* approach we utilise MotEvo [12], a probabilistic method which quantifies binding probabilities between nucleotide sequences and distinct motifs (in PWM format). MotEvo parameters were set to: *MotEvo_bg_binding_prior*, which is a prior for background binding probability, as 0.99 (which corresponds to an expectation of 1 site every 100 bp), a minimum binding posterior probability to consider a binding event (*MotEvo_min_binding_posterior*) as 0.01, and the Markov order of the background model (*MotEvo_Markov_chain_order*) to 1. In this case the output sitecount matrices contain summed posterior probabilities for each RBP binding to each region. Regardless of the approach selected the resulting information is stored in a per-exon, per-motif matrix ($N_{e,m}$), for each window separately. Throughout the following study whenever we run MAPP in *kmer-based* mode we count all 3-mers, 4-mers and 5-mers. In case of *pwm-based* analyses we infer binding probabilities of pre-filtered subset of motifs deposited in the ATtRACT databse [13] which were provided through the configuration file by two fields: *PWM_directory* and *seqlogo_directory*.

2.6.2 CSM Module software tools and scripts workflow

In the MAPP code repository the CSM module is located under: *modules/CREATE_SITECOUNT_MATRICES*. The workflow consists of the following rules (see Figure S28 and Supplementary Fig. S29):

- **CSM_create_outdir** : creates top-level output directory to store all module results.
- **CSM_extract_window_coord_and_sequence** : extracts relative coordinates and sequences for each of the sliding windows around 3'SS, 5'SS and PAS.
- **CSM_prepare_MotEvo_parameters** : prepares a textfile with all MotEvo parameters in a format expected by the tool.

- **CSM_prepare_sequences_for_MotEvo** : prepares textfiles with sequences of all windows in a format expected by MotEvo.
- **CSM_MotEvo_analysis** : executes MotEvo tool to infer the binding posterior probabilities of distinct short sequence motifs in a PWM format to various sliding windows.
- **CSM_create_MotEvo_sitecount_matrices** : combines all per-motif MotEvo output files into per-window sitecount matrices in a TSV format.
- **CSM_create_kmer_sitecount_matrices** : generates all per-window sitecount matrices with raw k-mer counts (this rule is applied only in cases where MAPP is run in kmer-based mode and is executed instead of all steps related to MotEvo).
- **CSM_create_links_to_matrices** : creates hard links to the sitecount matrices with a unified name.
- **CSM_all** : final rule of the workflow which gathers the output of all previous processing steps.

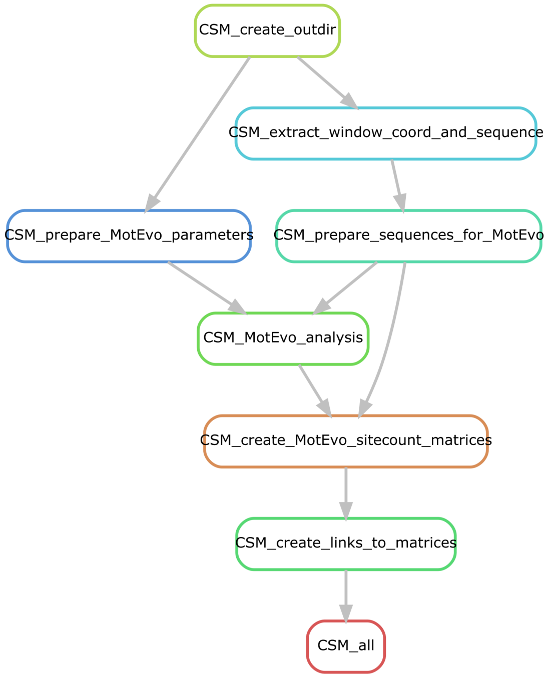

Figure S28. Snakemake rulegraph for the CSM module of MAPP executed in the pwm-based mode.

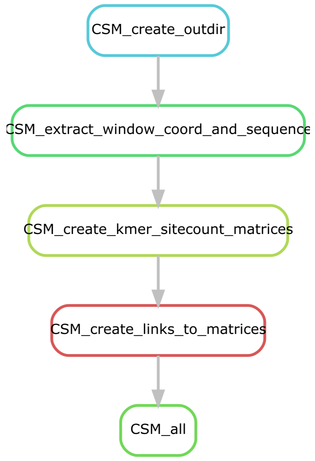

Figure S29. Snakemake rulegraph for the CSM module of MAPP executed in the kmer-based mode.

2.7 Modeling exons' inclusion (MAEI)

Snakemake rules which belong to this module are marked with a three-letter namespace: *MAE*.
The MAPP config file parameters relevant for the MAE module are presented in Figure S30.

2.7.1 MAE Module details

Our aim is to model previously quantified inclusion of cassette exons with the information stored in sitecount matrices (either raw counts of k-mers or binding probabilities for distinct PWMs). Note that the following procedure is applied to each of the sliding windows separately.

For every exon e we model its inclusion fraction $f_{e,s}$ in sample s with a logistic function $\Theta_{e,s}$:

$$f_{e,s} = \frac{i_{e,s}}{t_{e,s}} \sim \Theta_{e,s} = \frac{e^{b_s + c_e + N_{e,m} \times A_{m,s}}}{1 + e^{b_s + c_e + N_{e,m} \times A_{m,s}}}, \tag{2}$$

where $t_{e,s}$ is the total expression in sample s of the gene containing exon e, and $i_{e,s}$ the expression of transcripts containing exon e. The model parameters are the baseline inclusion level b_s of all exons in sample s, the baseline inclusion level c_e of exon e, the number of binding sites $N_{e,m}$ for motif m at exon e, and the activity $A_{m,s}$ of motif m in sample s. The motif activities $A_{m,s}$ are the key quantities of interest that account for the effect of short sequence motifs on the differential exon inclusion process and are inferred from the expression data, i.e. $i_{e,s}$ and $t_{e,s}$, and the computationally predicted site counts $N_{e,m}$. Note that, as specified, the model is redundant in that the motif activities $A_{m,s}$, exon inclusions c_e and sample inclusions b_s can be shifted so as to leave all probabilities $\Theta_{e,s}$ invariant. To remove this redundancy, we demand that the mean sample inclusion b_s is zero, i.e. $\sum_s b_s = 0$, and that the mean activity of each motif m is zero across the samples, i.e. $\sum_s A_{m,s} = 0$ for each m.
If we had observed $i_{e,s}$ exon inclusion transcripts out of a total $t_{e,s}$ for each exon e in each sample s, then given the model M (as parametrized by the logistic functions $\Theta_{e,s}$) the probability of observing all the quantified data obtained from the RNA-Seq experiment would simply equal a product of consecutive Bernoulli trials where exon inclusion is treated as "success" and exclusion as "failure":

$$P(D|M) = \prod_{s,e} \Theta_{e,s}^{i_{e,s}} \times (1 - \Theta_{e,s})^{t_{e,s} - i_{e,s}} \tag{3}$$

Using this and noting that $i_{e,s} = t_{e,s} f_{e,s}$ we can write the log-likelihood as

$$LL = \sum_{s,e} t_{e,s} \times \left[f_{e,s} \times (b_s + c_e + N_{e,m} \times A_{m,s}) - \log(1 + e^{b_s + c_e + N_{e,m} \times A_{m,s}}) \right], \tag{4}$$

which clearly brings out that the 'weight' of exon e in sample s in the fitting is simply given by the total number of transcripts $t_{e,s}$ of exon e in sample s. However, in general we cannot meaningfully estimate such absolute transcript numbers, i.e. the estimated total expression levels $t_{e,s}$ are only proportional to these observed transcript numbers, with an unknown proportionality constant. In addition, given that absolute expression levels of transcripts typically vary over $4-5$ orders of magnitude in RNA-seq, the likelihood (4) will be dominated by the fitting of the most highly expressed genes, which is clearly not desirable. To address both these issues, we will replace $t_{e,s}$ with renormalized expression level $R_{e,s}$ that is directly proportional to $t_{e,s}$ at low expression levels but saturates to a constant at expression levels significantly above a critical level t_s^{crit}, so that the weight of the highest expressed genes in the fitting remains limited. In particular, we define

$$R_{e,s} = C_s \frac{t_{e,s}}{t_{e,s} + t_s^{crit}} \tag{5}$$

where the pre-factor C_s is set so that the sum of all expression levels $\sum_{e,s} t_{e,s}$ remains invariant, i.e.

$$C_s = \frac{\sum_e t_{e,s}}{\sum_e \frac{t_{e,s}}{t_{e,s} + t_s^{\text{crit}}}}. \tag{6}$$

The parameter t_s^{crit} determines at what expression level $R_{e,s}$ starts to saturate and we chose to set t_s^{crit} to the median of the $t_{e,s}$ across all exons by default.

In a number of applications, such as knock-down experiments of RNA processing factors or other perturbations that are mostly targeted toward RNA processes such as splicing, there often is relatively little change in the absolute expression levels $t_{e,s}$ across the samples. In those cases it can be desirable to replace the sample-dependent statistical weights $R_{e,s}$ with sample-averaged weights R_e. This ensures that observations on the inclusion frequency of a given exon e are weighted equally across all samples s. Although sample-dependent weights $R_{e,s}$ (as defined above) are utilised with default settings of MAEI, the user can also specify to use sample-independent weights R_e that are useful when running on experiments where absolute levels $t_{e,s}$ vary relatively little. We define

$$R_e = C \frac{\langle t_e \rangle}{\langle t_e \rangle + t^{crit}}, \tag{7}$$

where $\langle t_e \rangle$ is the average expression of exon e across all samples and the factor C is given by:

$$C = \frac{\sum_e \langle t_e \rangle}{\sum_e \frac{\langle t_e \rangle}{\langle t_e \rangle + t^{crit}}}, \tag{8}$$

Finally, we can express the log-likelihood of our model as:

$$LL = \sum_{s,e} R \times [f_{e,s} \times (b_s + c_e + N_{e,m} \times A_{m,s}) - \log(1 + e^{b_s + c_e + N_{e,m} \times A_{m,s}})], \tag{9}$$

with R being set to either $R_{e,s}$ (default) or R_e as defined above.

We find maximum likelihood estimates of the model parameters using an EM algorithm where we iteratively calculate partial derivatives with respect to model parameters ($\frac{\partial LL}{\partial A_s}, \frac{\partial LL}{\partial b_s}, \frac{\partial LL}{\partial c_e}$), update their values

and re-calculate the likelihood until it converges. In order to ensure a successful procedure, we demand that at each iteration of the algorithm $\sum_s A_{m,s} = 0$ and $\sum_s b_{m,s} = 0$. Having enforced such constraints we let all parameters c_e adjust accordingly to preserve the likelihood at it's current value.

We obtain standard deviations of motif activities ($A_{m,s}$) from the Hessian matrix of the log-likelihood function at its optimum. Its negative inverse is an estimator of the covariance matrix of the model parameters. We use these estimates to standardize the activities: for every sample s we calculate a per-sample motif activity z-score: $Z_{m,s} = \frac{A_{m,s}}{\sigma_{m,s}}$.

In order to distinguish motifs with statistically significant z-scores from those with z-scores expected under a Gaussian background model, we fit the distribution of observed z-scores to a mixture of a uniform (foreground) and Gaussian (background) distribution:

$$P(D|M) = \rho \times \frac{1}{\max Z_{m,s} - \min Z_{m,s}} + (1-\rho) \times \frac{1}{\sqrt{2\pi}\sigma} \times e^{\frac{-(Z_{m,s}-\mu)^2}{2\sigma^2}}, \tag{10}$$

where the max and min functions are over all motifs m for a given sample s.
We find the maximum likelihood estimates for the parameters (ρ, μ, σ) of the model using an EM algorithm and then use the fitted parameters of the Gaussian background distribution, i.e. μ and σ, to 'renormalize' the z-scores as $Z^{\#}_{m,s} = \frac{Z_{m,s}-\mu}{\sigma}$. After this, we then finally transform these into p-values using these z-scores derive from a standard normal distribution. In order to assess statistical significance at $\alpha = 0.05$ level we also apply a Bonferroni correction where we adjust by the total number of motifs. In this way we obtain p-values $p_{m,s}$ for every motif m in every sample s (separately for every sliding window).

MAEI module may be parametrized by the configfile fields: *analysis_design_table* (as described in the previous modules), *min_motif_fraction* (minimal fraction of sites required to have a binding site for a given motif), *min_transcript_expression* (cutoff for the minimal total expression of all transcripts which could have included a given cassette exon), *average_expressions* (whether to utilise sample-averaged weights R_e, as described above) as well as *sorting_strategy* (sorting strategy for the output table, described in the "Analysis summary" subsection).

```
analysis_design_table: ""
min_motif_fraction: 0.01
min_transcript_expression: 1
average_expressions: False
sorting_strategy: "avg"
```

Figure S30. MAPP config file parameters for the MAE module

2.7.2 MAE Module software tools and scripts workflow

In the MAPP code repository the MAE module is located under: *modules/MAEI*.
The workflow consist of the following rules (see Figure S31):

- **MAE_create_outdir** : creates top-level output directory to store all module results.
- **MAE_prepare_inclusion_table** : filters out all cassette exons located on genes which total expression did not pass a minimal TPM threshold in all samples.
- **MAE_fit_model_parameters** : for each window separately for both 3'SS and 5'SS: fits the statistical model with motif activities driving cassette exon inclusion.
- **MAE_calculate_Zscores** : for each window separately for both 3'SS and 5'SS: calculates z-scores of motif activities.

- **MAE_extend_result_tables** : extracts the region IDs and motif IDs into separate columns of the motif activity tables.
- **MAE_calculate_Zscores_statistical_significancy** : runs a mixture model to assess statistical significance of the inferred motif activity z-scores.
- **MAE_collapse_results_for_region** : collapses all motif activity tables over distinct sliding windows into one TSV-formatted file (executed for both 3'SS and 5'SS separately).
- **MAE_all** : final rule of the workflow which gathers the output of all previous processing steps.

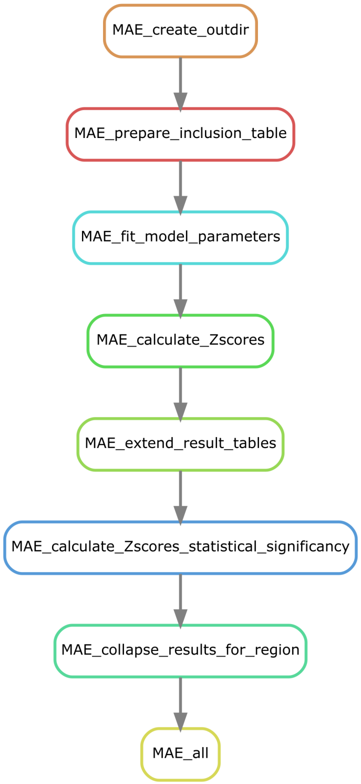

Figure S31. Snakemake rulegraph for the MAE module of MAPP.

2.8 Modeling poly(A) site usage (KAPACv2.0)

Snakemake rules which belong to this module are marked with a three-letter namespace: *KPC*. The MAPP config file parameters relevant for the KPC module are presented in Figure S32.

2.8.1 KPC Module details

KAPACv2.0, standing for K-mer Activity on PolyAdenylation site Choice version 2.0, builds upon our previously published KAPAC approach [10], whereas for the needs of MAPP we have implemented a new version of KAPAC, KAPACv2.0, which does not depend on the definition of sample contrasts, such as tumor versus normal, but can be applied to any set of samples. Another new features of KAPACv2.0 is that it is capable of running on both raw k-mer counts or binding sites predicted from position weight

matrices, similar to the MAEI model. That is, for a given sequence window relative to a poly(A) site p, KAPACv2.0 considers either the sum of site probabilities predicted with a PWM representing a motif k or the raw counts of a k-mer k. It uses then these counts ($N_{p,k}$) to model the relative usage $U_{p,s}$ of each poly(A) site p in sample s as follows (further details are provided in ref. [10]):

$$\log_2(U_{p,s}) = N_{p,k} * A_{k,s} + p_s + p_{s,e} + \epsilon, \tag{11}$$

whereas $c_{s,e}$ is the mean $\log_2$ relative usage of the poly(A) site p from exon e in sample s, c_s is the mean $\log_2$ relative usage of poly(A) site p across all samples, ϵ is the residual error, and the relative usage $U_{p,s}$ of a poly(A) site p from a terminal exon with I poly(A) sites in sample s is calculated from its usage $R_{p,s}$ as follows:

$$U_{p,s} = \frac{R_{p,s}}{\sum_{i=1}^{I} R_{i,s}} \tag{12}$$

KAPACv2.0 solves for the unknown activity $A_{k,s}$ of PWM / k-mer k in sample s and the corresponding error $\sigma_{k,s}$ using a least squares approach. Similar to the MAEI model (see above), KAPACv2.0 calculates then for every activity $A_{k,s}$ of PWM / k-mer k in sample s and its corresponding error $\sigma_{k,s}$ the z-score $z = \frac{A_{k,s}}{\sigma_{k,s}}$ and performs background correction as done for the MAEI z-scores (see above).

The KPC module may be parametrized by the configfile fields: *analysis_design_table* (as described in the previous modules), *window_size* (as described in the CSM module), *min_motif_fraction* (minimal fraction of sites required to have a binding site for a given motif in order to be considered), *unique_region_upstream_pas* (length of the regions upstream of the PAS used to consider them as overlapping with others), *unique_region_downstream_pas* (length of the regions downstream of the PAS used to mark them as overlapping with others) as well as *sorting_strategy* (sorting strategy for the output table, described in the "Analysis summary" subsection).

```
analysis_design_table: ""
window_size: 50
min_motif_fraction: 0.01
unique_region_upstream_pas: 200
unique_region_downstream_pas: 200
sorting_strategy: "avg"
```

Figure S32. MAPP config file parameters for the KPC module

2.8.2 KPC Module software tools and scripts workflow

In the MAPP code repository the KPC module is located under: *modules/KAPAC*.
The workflow consists of the following rules (see Figure S33):

- **KPC_create_outdir** : creates the top-level output directory to store all module results.
- **KPC_create_polyA_site_to_exon_mapping** : generates a text file with mapped poly(A) sites to their respective terminal exons.
- **KPC_tag_overlapping_polyA_sites** : annotates which of the poly(A) sites do not overlap with any other taking into account a given margin around a site.
- **KPC_select_pas_expression_columns** : filters the poly(A) sites expression table in order to retain only columns with the TPM values.
- **KPC_run_KAPAC_model** : fits for each window separately the KAPACv2.0 model.

- **KPC_extend_result_tables** : extracts the region IDs and motif IDs into separate columns of the motif activity tables.
- **KPC_calculate_Zscores_statistical_significancy** : runs a mixture model to assess statistical significance of the inferred motif activity z-scores.
- **KPC_collapse_results** : merges the KAPACv2.0 motif activity tables obtained for different windows into one TSV file.
- **KPC_all** : final rule of the workflow which gathers the output of all previous processing steps.

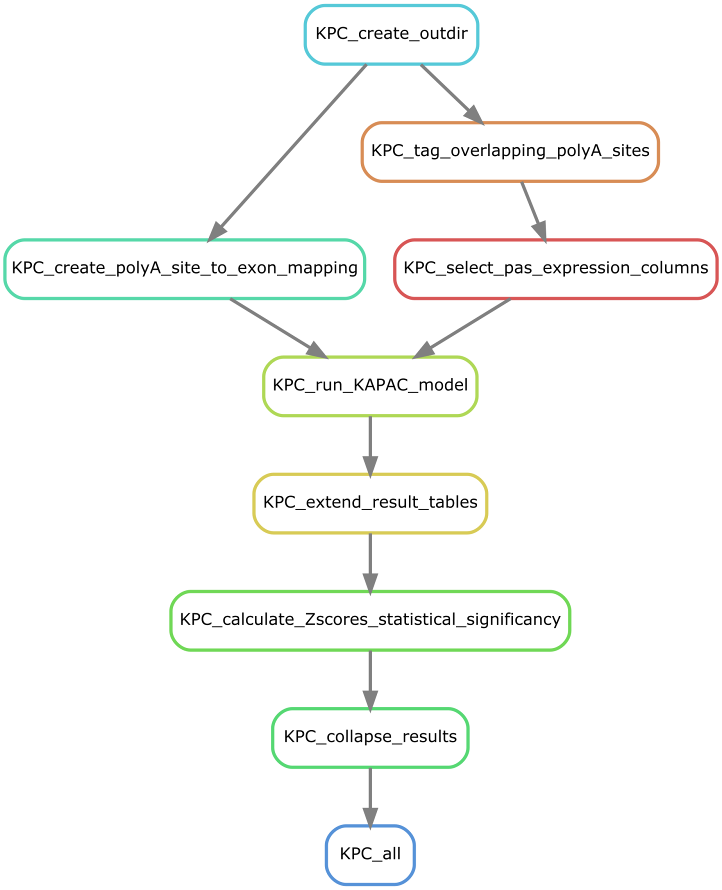

Figure S33. Snakemake rulegraph for the KPC module of MAPP.

2.9 Analysis summary

Snakemake rules which belong to this module are marked with a three-letter namespace: *RES*. The MAPP config file parameters relevant for the RES module are presented in Figure S34.

2.9.1 RES Module details

Following both statistical models and having inferred motif activities ($A_{m,s}$), their z-scores ($Z_{m,s}$) and p-values ($p_{m,s}$) in all samples and in every window we proceed to summarize the analysis, select those

results which we consider statistically significant and visualise them with heatmaps of activity z-scores, which we refer to as 'Impact Maps'. In the last module of the workflow we implemented two distinct strategies to filter motifs based on statistical significance, applicable to different analyses types (provided as fields in the configuration file *sorting_strategy*). In the *avg* mode we sort the results tables by a z-like statistic calculated over the set of all samples S as below:

$$Z_m = \sqrt{\frac{1}{S}\sum_{s=1}^{S} Z_{m,s}^2} \tag{13}$$

We call a given motif m statistically significant if and only if there exists a window w within which for at least half of the samples $p_{m,s}^{w}$ are below a previously defined cutoff (configfile parameter *max_pval* for which we always used 0.05). This strategy is designed for common comparative analyses of two biological conditions, each being sequenced in multiple replicates. The other approach - *max* - requires m to be called as statistically significant in only one sample in order to annotate significance to the whole motif. In that approach we also sort motifs in the result tables by the magnitude of the smallest Bonferroni-corrected p-value (selected across all samples). The rationale behind this strategy was to provide an insightful way of investigating datasets which consist of multiple distinct conditions. For every motif called as statistically significant we plot an Impact Map as a visual summary of that motifs activity on exon inclusion and poly(A) site usage. These plots require sample-specific information and that is why the previously mentioned *analysis_design_table* is a parameter for the RES module too.

```
analysis_design_table: ""
max_pval: 0.05
sorting_strategy: "avg"
```

Figure S34. MAPP config file parameters for the RES module

2.9.2 RES Module software tools and scripts workflow

In the MAPP code repository the RES module is located under: *modules/REPORT_RESULTS*.
The workflow consist of the following rules (see Figure S35):

- **RES_create_outdir** : creates top-level output directory to store all module results.
- **RES_annotate_statistical_significance** : appends one additional column to the motif activity tables which denotes whether we consider the activity z-score of a given motif in a given window as statistically significant.
- **RES_select_top_motifs** : selects all motifs which were considered as statistically significant around 3'SS, 5'SS and PAS.
- **RES_prepare_Zscores_tables** : prepares TSV-formatted tables with per-sample activity z-scores for statistically significant motifs (over all windows of 3'SS, 5'SS and PAS).
- **RES_plot_Zscores_heatmaps** : generates per-motif MAPP Impact Maps with z-scores of motif activities.
- **RES_all** : final rule of the workflow which gathers the output of all previous processing steps.

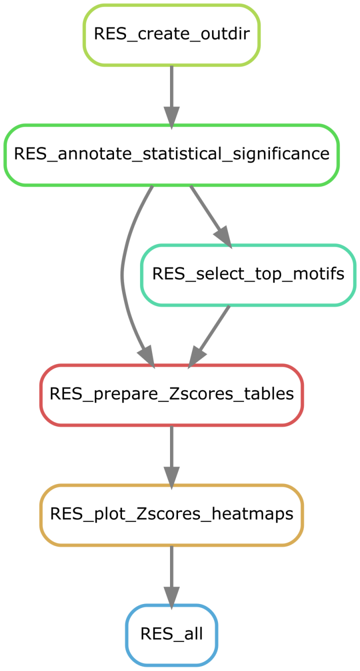

Figure S35. Snakemake rulegraph for the RES module of MAPP.

2.10 Final MAPP Report

At the end of the workflow we designed a few additional steps which prepare an output directory with the most important text files and tables as well as a summary report in HTML format. Summary directory is also compressed into .tar.gz format to facilitate reproducibility and usability: users may exchange their results as well as run configurations easily. The final HTML report contains a sorted table of motifs whose activity z-scores were reported to be statistically significant in at least one sliding window around any of the processing sites. The columns of that table include: motif ID, sequence logo (in case of a PWM-based MAPP execution), three maximum activity z-scores (with the maximum taken over all sliding windows around each of the analyzed sites: 3'SS, 5'SS, PAS), ranking score (set to an average of the three aforementioned z-scores) and the Impact Map.

Top-level Snakemake rules related to the final summary are marked with a four-letter namespace: *MAPP*.

2.11 Transcripts' stability effect analysis

In order to provide insights into whether some of the KAPACv2.0 results might be (partly) driven by changes in mRNA stability we have conducted the following analysis: First, based on the ENSEMBL gene annotation (GTF format) we have selected terminal exons of all protein coding transcripts and intersected their 3' end coordinates (+/- 25 nt) with the genomic coordinates of experimentally observed poly(A) sites as provided by the PolyASite atlas [14] and retained only terminal exons whose 3' end is supported by an experimentally observed poly(A) site. Importantly, we excluded terminal exons that intersected with more than one experimentally observed poly(A) site as well as terminal exons that intersected with any other terminal exon of protein-coding transcripts (as specified in the ENSEMBL gene annotation) in order to remove from our analysis all transcripts that might undergo 3' UTR shortening / lengthening due to the usage of alternative poly(A) sites. From those terminal exons which were left, we further

selected only those that had exactly one STOP codon annotated. For example, if a terminal exon had two distinct transcripts having two different STOP codons annotated, the terminal exon was dropped as well. Finally, we ended up with a set of 3'UTR coordinates located within terminal exons, that do not overlap with any other terminal exon and whose 3' end has experimental support. As RNA-seq data are biased towards having depleted read coverage at the 3' end of transcripts we considered only 3'UTRs with a minimum length of 200 nt. We utilised MAPP's CSM module to create k-mer based sitecount matrices for all remaining 3'UTRs. Transcript expression levels (quantified with Salmon) were collapsed for transcripts sharing the same terminal exon in order to obtain terminal exon centered expression levels, whereas we excluded all terminal exons having an expression level below 1 TPM in at least one sample from our downstream analysis. Finally, we determined the average expression across the control samples of a particular experiment and then calculated the log2 fold-changes of each of the treated samples (e.g. knock-down, overexpression) to this average. In the end, for each treated sample we compare the log2 fold-changes of the following two groups: (i) terminal exons that contain a specific k-mer within their 3'UTR as well as (ii) terminal exons that have zero-counts for the k-mer. We make use of a two-tailed rank-sum test to assess whether the log2 fold-changes differ between the two groups, which would imply that the presence of a given k-mer in 3'UTRs impacts the expression level of the corresponding transcripts. Results of this analysis for a microRNA (as a positive control) as well as two RBP experiments are presented in Figure S15.

2.12 Gene ontology analysis

Prior to gene ontology (GO) analysis, differentially expressed cassette exons were obtained by performing t-tests of normal brain vs. glioblastoma samples using R [15] (v.4.2.0) and "matrixTests" library (v.0.1.9.1). P-values were adjusted for multiple comparisons using Benjamini-Hochberg (BH) method. Genes containing differentially expressed cassette exons with binding sites (binding probability > 0.6) for the PTBP1 and the RBFOX RBPs within the corresponding regions inferred by MAPP. For the PTBP1 RBP 3'SS the considered windows reached from -125 to +50 nt and for the 5'SS there was only one window considered from -50 to 0 nt. For the RBFOX RBPs 5'SS the considered regions reached from 0 to +200 nt. The exons having counts in both of the windows were used for gene ontology (GO) analysis which was performed with the "enrichGO" function from the Bioconductor package "clusterProfiler" [16] (v.4.4.1) for each of the GO categories: biological process (BP), molecular function (MF) and cellular component (CC). As minimal number of genes annotated per ontology term (minGSSize) we used 10 and an adjusted p-value cutoff (pvalueCutoff) for enrichment tests was 0.1. P-values for enriched GO terms were adjusted for multiple comparisons using BH method. All genes having MAPP quantified cassette exons in the dataset served as a background ("universe") for GO analysis. The HTML-report was generated with the "Bookdown" R package [17] (v.0.29) and can be found in the supplementary materials package uploaded to the Zenodo server.

2.13 ENCODE MAPP results clustering

ENCODE screening results were curated such that from every results directory of an ENCODE experiment the top three k-mers with the highest impact on 3'SS, 5'SS or PAS, respectively, were selected and clustered into two clusters using the clustering function implemented in the SMEAGOL package [18]. In case only two k-mers were available, these were clustered into one cluster; experiments with only one or no significant k-mers were dropped. In the former case if the top-ranked k-mer was not clustered with any other k-mer the experiments were dropped as well, similarly if the intra-cluster similarity was below 0.75 for the cluster consisting of two k-mers. Additionally, experiments were removed if not all sequencing samples passed the quality filtering criteria. Finally, only experiments having a statistically significant k-mer activity in at least 3 out of 4 samples within at least one of the analysis windows were retained. From the curated list of ENCODE experiments the k-mers with the highest impact on 3'SS, 5'SS or

PAS, respectively, were selected (resulting in three tables). For each table separately, the k-mers of the table were clustered into a distinct number of clusters such that the minimum intra-cluster similarity was ≥ 0.75. In Figure S5 clusters are shown that have for no RBP a contradicting motif in the curated ATtRACT database (i.e. the ATtRACT motif and the top k-mer have a similarity smaller than 0.25), unless another RBP has a more substantial support (i.e. the ATtRACT motif and the top k-mer have a minimum similarity of 0.5), in which case only the contradicting experiment was removed. Finally, clusters consisting of only one k-mer were removed as well. All clusters and the detailed processing are available in the supplementary materials package on Zenodo.

References

1. Martin, M. Cutadapt removes adapter sequences from high-throughput sequencing reads. *EMBnet. journal* **17**, 10–12 (2011).

2. Dobin, A. *et al.* Star: ultrafast universal rna-seq aligner. *Bioinformatics* **29**, 15–21 (2013).

3. Li, H. *et al.* The sequence alignment/map format and samtools. *Bioinformatics* **25**, 2078–2079 (2009).

4. DeLuca, D. S. *et al.* Rna-seqc: Rna-seq metrics for quality control and process optimization. *Bioinformatics* **28**, 1530–1532 (2012).

5. Wang, L., Wang, S. & Li, W. Rseqc: quality control of rna-seq experiments. *Bioinformatics* **28**, 2184–2185 (2012).

6. Andrews, S. *et al.* FastQC. Babraham Institute (2012).

7. Trincado, J. L. *et al.* Suppa2: fast, accurate, and uncertainty-aware differential splicing analysis across multiple conditions. *Genome biology* **19**, 1–11 (2018).

8. Herrmann, C. J. *et al.* Polyasite 2.0: a consolidated atlas of polyadenylation sites from 3 end sequencing. *Nucleic acids research* **48**, D174–D179 (2020).

9. Patro, R., Duggal, G., Love, M. I., Irizarry, R. A. & Kingsford, C. Salmon provides fast and bias-aware quantification of transcript expression. *Nature methods* **14**, 417–419 (2017).

10. Gruber, A. J. *et al.* Discovery of physiological and cancer-related regulators of 3 utr processing with kapac. *Genome biology* **19**, 1–17 (2018).

11. Dale, R. K., Pedersen, B. S. & Quinlan, A. R. Pybedtools: a flexible python library for manipulating genomic datasets and annotations. *Bioinformatics* **27**, 3423–3424 (2011).

12. Arnold, P., Erb, I., Pachkov, M., Molina, N. & van Nimwegen, E. Motevo: integrated bayesian probabilistic methods for inferring regulatory sites and motifs on multiple alignments of dna sequences. *Bioinformatics* **28**, 487–494 (2012).

13. Giudice, G., Sánchez-Cabo, F., Torroja, C. & Lara-Pezzi, E. Attract—a database of rna-binding proteins and associated motifs. *Database* **2016** (2016).

14. Herrmann, C. J. *et al.* PolyASite 2.0: a consolidated atlas of polyadenylation sites from 3 end sequencing. *Nucleic Acids Research* **48**, D174–D179 (2019). URL `https://doi.org/10.1093/nar/gkz918`. `https://academic.oup.com/nar/article-pdf/48/D1/D174/31697497/gkz918.pdf`.

15. R Core Team. *R: A Language and Environment for Statistical Computing.* R Foundation for Statistical Computing, Vienna, Austria (2022). URL `https://www.R-project.org/`.

16. Yu, G., Wang, L.-G., Han, Y. & He, Q.-Y. clusterprofiler: an r package for comparing biological themes among gene clusters. *OMICS: A Journal of Integrative Biology* **16**, 284–287 (2012).

17. Xie, Y. *bookdown: Authoring Books and Technical Documents with R Markdown* (Chapman and Hall/CRC, Boca Raton, Florida, 2016). URL `https://bookdown.org/yihui/bookdown`. ISBN 978-1138700109.

18. Lal, A., Galvao Ferrarini, M. & Gruber, A. J. Investigating the human hostmdash;ssrna virus interaction landscape using the smeagol toolbox. *Viruses* **14** (2022). URL `https://www.mdpi.com/1999-4915/14/7/1436`.
