# Supplementary material for: MAPP unravels frequent co-regulation of splicing and polyadenylation by RNA-binding proteins and their dysregulation in cancer
Source: Nat Commun. 2024 May 15;15:4110. doi: 10.1038/s41467-024-48046-1 (PMC11096328; doi:10.1038/s41467-024-48046-1)
Supplement: Supplementary file 10 — Reporting Summary [file 41467_2024_48046_MOESM10_ESM.pdf]

nature portfolio

Corresponding author(s): Andreas J. Gruber
Last updated by author(s): Mar 26, 2024

Reporting Summary

Nature Portfolio wishes to improve the reproducibility of the work that we publish. This form provides structure for consistency and transparency in reporting. For further information on Nature Portfolio policies, see our Editorial Policies and the Editorial Policy Checklist.

Statistics

For all statistical analyses, confirm that the following items are present in the figure legend, table legend, main text, or Methods section.

n/a	Confirmed	
☐	☒	The exact sample size (*n*) for each experimental group/condition, given as a discrete number and unit of measurement
☒	☐	A statement on whether measurements were taken from distinct samples or whether the same sample was measured repeatedly
☐	☒	The statistical test(s) used AND whether they are one- or two-sided<br>*Only common tests should be described solely by name; describe more complex techniques in the Methods section.*
☒	☐	A description of all covariates tested
☐	☒	A description of any assumptions or corrections, such as tests of normality and adjustment for multiple comparisons
☐	☒	A full description of the statistical parameters including central tendency (e.g. means) or other basic estimates (e.g. regression coefficient) AND variation (e.g. standard deviation) or associated estimates of uncertainty (e.g. confidence intervals)
☐	☒	For null hypothesis testing, the test statistic (e.g. *F*, *t*, *r*) with confidence intervals, effect sizes, degrees of freedom and *P* value noted<br>*Give P values as exact values whenever suitable.*
☒	☐	For Bayesian analysis, information on the choice of priors and Markov chain Monte Carlo settings
☒	☐	For hierarchical and complex designs, identification of the appropriate level for tests and full reporting of outcomes
☐	☒	Estimates of effect sizes (e.g. Cohen's *d*, Pearson's *r*), indicating how they were calculated

Our web collection on statistics for biologists contains articles on many of the points above.

Software and code

Policy information about availability of computer code

Data collection	Download: curl, version 7.77.0<br>Analysis: https://zenodo.org/doi/10.5281/zenodo.10845501 and https://zenodo.org/doi/10.5281/zenodo.5789986
Data analysis	MAPP, version 1.0.0<br>python version 3.10.8<br>pyyaml, version 6.0<br>pybedtools, version 0.9.0<br>biopython, version 1.79<br>pandas, version 1.4.2<br>scipy, version 1.7.3<br>matplotlib, version 3.5.2<br>seaborn, version 0.11.2<br>bedtools, version 2.30.0<br>samtools, version 1.3<br>jupyterlab, version 3.5.2<br>smeagol, version 0.1.1<br>rpy2, version 3.5.1

For manuscripts utilizing custom algorithms or software that are central to the research but not yet described in published literature, software must be made available to editors and reviewers. We strongly encourage code deposition in a community repository (e.g. GitHub). See the Nature Portfolio guidelines for submitting code & software for further information.

Data

Policy information about availability of data

All manuscripts must include a data availability statement. This statement should provide the following information, where applicable:

- Accession codes, unique identifiers, or web links for publicly available datasets
- A description of any restrictions on data availability
- For clinical datasets or third party data, please ensure that the statement adheres to our policy

The results generated in this study have been deposited in the Zenodo database under accession code 5789986 [https://doi.org/10.5281/zenodo.5789986].

The raw RNA-Seq data are available at the following records:
GSE56010 [https://www.ncbi.nlm.nih.gov/geo/query/acc.cgi?acc=GSE56010]
GSE69656 [https://www.ncbi.nlm.nih.gov/geo/query/acc.cgi?acc=GSE69656]
GSE71468 [https://www.ncbi.nlm.nih.gov/geo/query/acc.cgi?acc=GSE71468]
GSE179630 [https://www.ncbi.nlm.nih.gov/geo/query/acc.cgi?acc=GSE179630]
PRJNA798408 [https://www.ebi.ac.uk/ena/browser/view/PRJNA798408]
GSE204705 [https://www.ncbi.nlm.nih.gov/geo/query/acc.cgi?acc=GSE204705]
GSE185861 [https://www.ncbi.nlm.nih.gov/geo/query/acc.cgi?acc=GSE185861]
GSE147352 [https://www.ncbi.nlm.nih.gov/geo/query/acc.cgi?acc=GSE147352]
GSE38805 [https://www.ncbi.nlm.nih.gov/geo/query/acc.cgi?acc=GSE38805]
PRJEB4337 [https://www.ebi.ac.uk/ena/browser/view/PRJEB4337]

Additionally we used ENSEMBL annotation, version 38, ATtRACT database (https://attract.cnic.es/) and Poly(A) site Atlas 2.0 (https://polyasite.unibas.ch/).

Research involving human participants, their data, or biological material

Policy information about studies with human participants or human data. See also policy information about sex, gender (identity/presentation), and sexual orientation and race, ethnicity and racism.

Reporting on sex and gender	Sex was not considered in this study. Only samples available from other studies / cohorts that were previously already finalized / published were considered. Used samples are listed in Supp. Data 1, 2, and 3.
Reporting on race, ethnicity, or other socially relevant groupings	Race, ethnicity and socially relevant groupings were not considered in this study. Only samples available from other studies / cohorts that were previously already finalized / published were considered. Used samples are listed in Supp. Data 1, 2, and 3.
Population characteristics	Population characteristics were not considered in this study. Only samples available from other studies / cohorts that were previously already finalized / published were considered. Used samples are listed in Supp. Data 1, 2, and 3.
Recruitment	Only samples available from other studies / cohorts that were previously already finalized / published were considered. Used samples are listed in Supp. Data 1, 2, and 3. Details of the collection of samples can be found in the studies that initially published the data.
Ethics oversight	Only samples available from other studies / cohorts that were previously already finalized / published were considered. Used samples are listed in Supp. Data 1, 2, and 3. Ethics oversight may be found in the studies that initially published the data.

Note that full information on the approval of the study protocol must also be provided in the manuscript.

Field-specific reporting

Please select the one below that is the best fit for your research. If you are not sure, read the appropriate sections before making your selection.

☒ Life sciences ☐ Behavioural & social sciences ☐ Ecological, evolutionary & environmental sciences

For a reference copy of the document with all sections, see nature.com/documents/nr-reporting-summary-flat.pdf

Life sciences study design

All studies must disclose on these points even when the disclosure is negative.

Sample size	All used data including the exact number of samples and the corresponding accession numbers / sample ids are available in Supp. Data 1, 2, and 3.
Data exclusions	RNA sequencing data of RNA-binding protein knock-down and control samples were selected as described in the Methods and Supplementary Information. GBM samples presented in the figures of the main manuscript were used as selected and described by a previous study (https://doi.org/10.1186/s13059-018-1415-3). From the RNA sequencing samples of the human protein atlas tissues salivary gland, pancreas and placenta were excluded. Generally, all RNA sequencing samples not fulfilling our applied quality measures/thresholds were excluded. Publicly available eCLIP data were selected according to availability and the needs of the study (details are provided in the Supplementary

Information).

Replication

Available technical replicates (as specified in the public datasets) were considered as long as they passed all our quality measures/thresholds (described in the Methods and Supplementary Information).

Randomization

Sample randomization was not applied, because in our study we have analyzed already created / published data sets, i.e. only samples available from already finalized / published studies were taken into account. Details of the sample collection and processing procedures can be found in the studies that initially collected and published the raw sequencing data.

Blinding

Only samples previously collected and processed by other studies / cohorts were considered. Given that in this study we have analyzed already created sequencing data, sample blinding was not relevant. Details of the collection and processing of samples can be found in the studies that have initially published the raw sequencing data.

Reporting for specific materials, systems and methods

We require information from authors about some types of materials, experimental systems and methods used in many studies. Here, indicate whether each material, system or method listed is relevant to your study. If you are not sure if a list item applies to your research, read the appropriate section before selecting a response.

Materials & experimental systems

n/a	Involved in the study
☒	☐ Antibodies
☒	☐ Eukaryotic cell lines
☒	☐ Palaeontology and archaeology
☒	☐ Animals and other organisms
☒	☐ Clinical data
☒	☐ Dual use research of concern
☒	☐ Plants

Methods

n/a	Involved in the study
☒	☐ ChIP-seq
☒	☐ Flow cytometry
☒	☐ MRI-based neuroimaging

Plants

Seed stocks

Report on the source of all seed stocks or other plant material used. If applicable, state the seed stock centre and catalogue number. If plant specimens were collected from the field, describe the collection location, date and sampling procedures.

Novel plant genotypes

Describe the methods by which all novel plant genotypes were produced. This includes those generated by transgenic approaches, gene editing, chemical/radiation-based mutagenesis and hybridization. For transgenic lines, describe the transformation method, the number of independent lines analyzed and the generation upon which experiments were performed. For gene-edited lines, describe the editor used, the endogenous sequence targeted for editing, the targeting guide RNA sequence (if applicable) and how the editor was applied.

Authentication

Describe any authentication procedures for each seed stock used or novel genotype generated. Describe any experiments used to assess the effect of a mutation and, where applicable, how potential secondary effects (e.g. second site T-DNA insertions, mosiacism, off-target gene editing) were examined.
